# Tailoring High‐Valent Iron‐Oxo Species Redox via Extended π‐Fe3d–O2p Orbital Overlapping Toward Chemical‐Efficient Water Purification

**DOI:** 10.1002/anie.5511823

**Published:** 2026-07-13

**Authors:** Hongyu Zhou, Junwen Chen, Jiuyi Wang, Xinhao Wang, Qiming Zhang, Shiying Ren, Xuning Li, Li Gao, Shaobin Wang, Xiaoguang Duan

**Affiliations:** ^1^ School of Chemical Engineering Adelaide University Adelaide South Australia Australia; ^2^ State Key Laboratory of Catalysis Dalian Institute of Chemical Physics Chinese Academy of Sciences Dalian P.R. China; ^3^ School of Science STEM College RMIT University Melbourne Victoria Australia

**Keywords:** conjugated organic framework, Fenton‐like reactions, high‐valent iron oxo species, low‐oxidant‐consumption, wastewater treatment

## Abstract

High‐valent iron oxo species (Fe(IV)) are attractive for wastewater treatment because of their high selectivity toward organic pollutants in complex water matrices, but their intrinsic redox properties drive rapid quenching by peroxide precursors, causing excessive chemical consumption. This work addresses this challenge by anchoring Fe(IV) on an iron‐phthalocyanine‐based conjugated organic framework (FePPC) featuring multi‐layered reticular structures and strong π−Fe3d−O2p orbital overlapping. The two‐dimensional planar structures provided easily accessible active sites and the extended in‐plane conjugation fine‐tunes the redox reactivity of surface‐confined Fe(IV) species, suppressing unproductive decay while preserving selectivity toward diverse pollutants, yielding a 3.2‐fold enhancement in Fe(IV) utilization efficiency. Combined experimental and computational results show that the enhanced orbital overlapping delocalizes electrons at the Fe(IV)═O bond and reduces occupancy of its anti‐bonding π* orbital, thereby strengthening the bond against nucleophilic attack by peroxymonosulfate, suppressing O_2_ evolution, and improving both pollutant selectivity and peroxide stoichiometric efficiency. When integrated into a scale‐up membrane reactor, FePPC achieved over 95% micropollutant removal during 72 h of continuous operation. This work fills an important knowledge gap in understanding Fe(IV) redox properties and selectivity, addressing technical bottlenecks in Fe(IV)‐based AOP systems toward low‐chemical consumption and high‐efficiency wastewater treatment.

## Graphical abstract

We address the long‐standing challenge of reactivity–selectivity trade‐off by precisely tuning the redox properties of Fe(IV), rather than simply accelerating its formation, to enable chemically efficient and low‐emission water treatment.

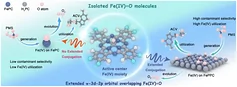

## 1 Introduction

High‐valent iron oxo species (HVI) have attracted increasing research interest in heterogeneous catalysis, including methane conversion [1], water oxidation [2], and contaminant degradation [3, 4, 5]. Compared with conventional reactive oxygen species (ROS) such as hydroxyl (HO^•^) and sulfate radicals (SO_4_^•−^), HVI feature an exceptional long lifetime (7 s in aqueous solutions vs. less than 40 µs for inorganic radicals) and particular selectivity to organic pollutants in real water matrices [6], while retaining a moderate oxidation capacity that enables diverse oxidation pathways, such as electron transfer, oxygen transfer, hydrogen abstraction, and electrophilic addition, outperforming the electron transfer pathway (ETP). In addition, iron precursors are cheap, nontoxic and Earth‐abundant, further highlighting the great potential of HVI in practical water and soil remediation. HVI can be generated via either homogeneous and heterogeneous pathways [7, 8]. Particularly, for heterogeneous catalysts (e.g., single‐atom catalysts [9, 10], the electronic structures of the iron center and its adsorption mode/strength toward peroxides could be elaborately regulated through coordination chemistry manipulation [11, 12, 13], cation intercalation [14], defect engineering [15, 16], and confinement effects [17], to achieve fast peroxide activation and oriented HVI (predominantly, Fe(IV)) generation.

Despite substantial efforts to regulate the generation rate and selectivity of Fe(IV), its intrinsic redox properties were largely overlooked in previous advanced oxidation process (AOP) studies. Distinct Fe(IV) species with fundamentally different redox properties are often ambiguously classified into a single group, leading to many contradictory conclusions in the literature. For example, aqueous Fe(IV), [Fe═O(H_2_O)_6_]^2+^, generated in a benchmark homogeneous Fe^2+^/perxoxydisulfate system, could be easily quenched by alcohols, while the heterogeneous Fe─N_4_‐based Fe(IV) is less reactive toward alcohol oxidation [18, 19]. Such diverse redox characteristic of Fe(IV) greatly differs from conventional ROS like radicals or singlet oxygen, thereby rendering Fe(IV) more flexible in contaminant oxidation. In addition, the knowledge gap in Fe(IV) characteristics across different systems can lead to inappropriate estimates and the use of secondary reaction rate constants when quantifying the contributions of different Fe(IV) species [20]. Generally, the reactivities of Fe(IV) are determined by the Fe(IV)═O configuration, the nature of peripheral coordinating ligands, and solvent properties, which regulate the interfacial chemical microenvironment and consequently the electronic/spin state of Fe(IV)═O in reactions. For example, Hastings et al. [21] localized the Fe(IV)═O unit within a rigid organic macrocycle, resulting in superior stability of Fe(IV) at ambient temperatures, with a half‐life of 21 h at 70°C in CH_3_CN. Hong et al. [22] found that the topology transformation of the two bidentate N‐ligands of Fe(IV) from *trans*‐position to *cis*‐position effectively enhanced the reactivity of Fe(IV) due to the altered orbital hybridization configuration.

Although highly reactive Fe(IV) species can promote rapid electron or oxygen transfer and effective oxidation of organic contaminants, excessive reactivity often induces undesired side reactions with Fe(II) and precursor peroxides (corresponding to the catalase activity), resulting in poor contaminant selectivity and low Fe(IV) utilization efficiency in Fenton‐like systems (corresponding to the peroxidase activity) [23]. For example, Fe(IV) coordinated with 1,4,8,11‐tetraaza[14]annulene molecules readily decomposed hydrogen peroxide into O_2_ without generating reactive species [24], while Fe(IV) coordinated with tetra‐amido‐macrocyclic ligand significantly inhibited direct hydrogen peroxide decomposition and retained high selectivity toward micropollutants due to the strong σ electron donation effects of amide groups [25]. Therefore, it is crucial to balance the trade‐off between catalase and peroxidase activity to achieve higher Fe(IV) selectivity toward contaminants. Moreover, the excessively reactive Fe(IV) may accelerate the decomposition of active sites, thereby compromising long‐term cyclic stability [26]. To date, regulation of Fe(IV) reactivity and stability has been limited primarily to nonaqueous homogeneous systems. In contrast, achieving selective Fe(IV) generation with modulated reactivity for selective contaminant degradation remains a critical bottleneck in heterogeneous Fe(IV)‐based AOPs systems [27].

Herein, we designed an iron‐phthalocyanine‐based conjugated organic framework (FePPC) as a molecular‐precision catalyst for efficient peroxymonosulfate (PMS) activation, enabling selective production of Fe(IV) and selective reaction pathways toward emerging water contaminants. Kinetic modeling results demonstrate the necessity of modulating Fe(IV) reactivity to suppress unproductive competition reactions and improve its utilization efficiency in pollutant oxidation. X‐ray photoelectron spectroscopy (XPS), x‐ray absorption near‐edge structure (XANES), extended x‐ray absorption fine structure (EXAFS), Mössbauer spectroscopy, spherical aberration‐corrected high‐resolution transmission electron microscopy (AC‐TEM), specific surface area (SSA) analysis, and Nyquist plot analysis suggest that FePPC, together with its monomer counterpart iron phthalocyanine (FePC), feature analogous chemical environment at the central iron site but distinct macroscopic structures and in‐plane conjugation degree, which provided much more accessible sites within layers for interacting with substrates and effectively regulated the reactivity of the Fe(IV)═O species. Electrochemical techniques, *cis*/*trans* selectivity toward stilbene, kinetic isotope effects and isotope tracing of both liquid and gas intermediates reveal that, despite the higher initial reaction rate for FePC, FePPC exhibits optimized reactivity against self‐decomposition and selectively proceeds pollutant oxidation, achieving high Fe(IV) utilization efficiency. Both quantum‐chemical and first‐principles theoretical calculations were conducted to reveal the degree of molecular substrate conjugation in modulating the electronic structure and, consequently, the reactivity with PMS to mitigate self‐decomposition. This work provides new insights into activity–selectivity relationships in AOPs to achieve an orchestrated decontamination process, offering a promising pathway toward sustainable and chemical‐efficient water treatment.

## 2 Results and Discussion

### 2.1 Synthesis and Characterizations

**FIGURE 1:**
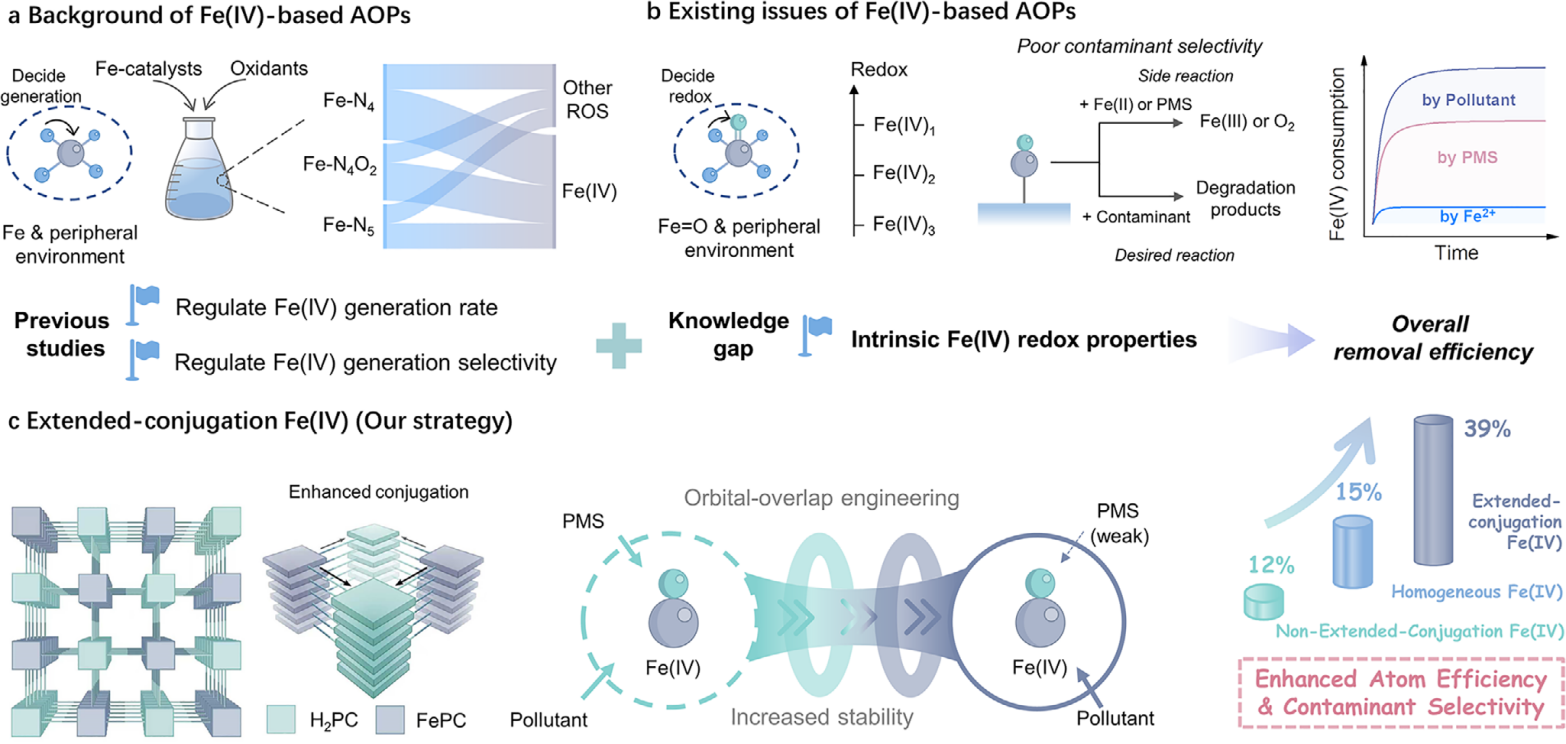
(a) Background of Fe(IV)‐based AOPs. Most previous researches focus on accelerating the generation of Fe(IV) or elevating the generation selectivity toward Fe(IV). (b) Issues faced by previous Fe(IV)‐based AOPs, i.e., competitive side reactions induced low chemical utilization efficiency. (c) Our extended‐conjugation strategy safeguards Fe(IV) from the nucleophilic attack of PMS and elevates the atom utilization efficiency of Fe(IV) and selectivity toward pollutants.

As shown in Figure 1, previous studies on Fe(IV)‐based AOPs mainly focused on enabling fast and selective Fe(IV) generation, while the intrinsic redox properties of Fe(IV) in different chemical environments were heavily overlooked. Particularly, due to the high oxidative capacity of Fe(IV), its rapid accumulation would not only accelerate contaminant degradation but also induce side reactions via directly reacting with iron and peroxide precursors. For example, in the benchmark homogeneous Fe^2+^/PMS system, kinetic modelling indicates that Fe^2+^ and PMS are both major sinks for Fe(IV), with only 34% of Fe(IV) effectively participating in the oxidation of organic pollutants (Figures S1 and S2; Table S1) [28]. In addition, increasing Fe^2+^ dosage from 0.05 to 0.8 mM leads to a decline in Fe(IV) utilization efficiency from 37% to 24% (Figure S3), while the highest effective PMS‐to‐Fe(IV) conversion efficiency is only 14% (Figure S4). Thus, the majority of PMS is decomposed by directly reacting with the generated Fe(IV) rather than contributing to pollutant oxidation. Although the Fe(IV) utilization efficiency in homogeneous Fe^2+^/PMS systems could not be quantitatively transferred to heterogeneous Fe(IV) systems, the results highlight the importance of modulating Fe(IV) redox to improve the chemical utilization efficiency of PMS.

**FIGURE 2:**
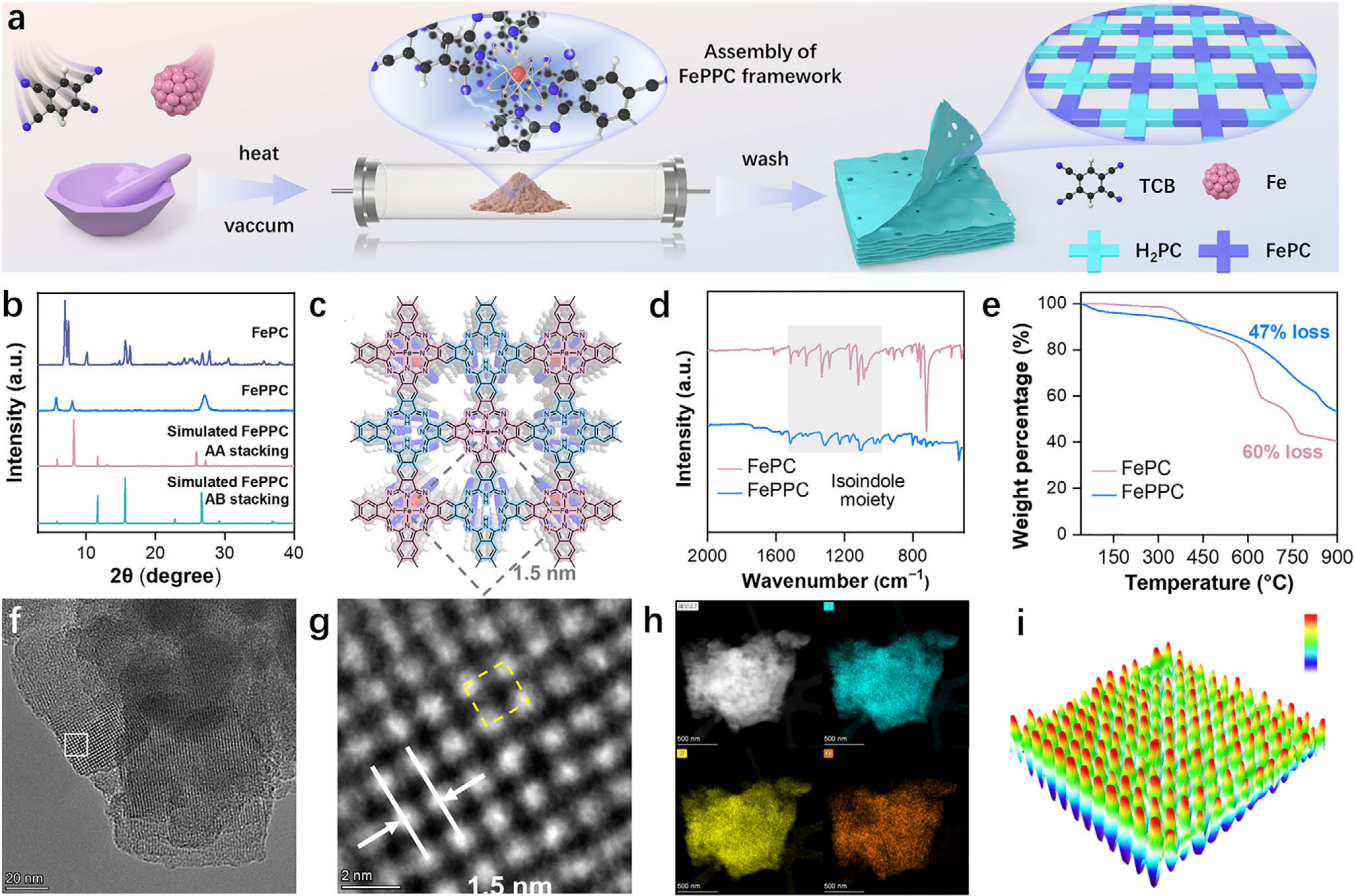
(a) Illustration of the assembly process of FePPC from tetracyanobenzene under thermal treatment. (b) XRD patterns of FePC and FePPC. (c) Top and side views of simulated FePPC structure. (d) FT‐IR spectra of FePC and FePPC. (e) TGA curves of FePC and FePPC. (f) Aberration corrected TEM images of FePPC. (g) High‐resolution TEM image and (h) corresponding element mapping of FePPC. (i) Atom‐overlapping Gaussian‐function‐fitting mapping derived from f.

Inspired by the capacity of conjugation to regulate the electronic structure and charge mobility of metal centers [29, 30, 31], we employed a flame‐sealed calcination strategy to construct two‐dimensional layered FePPC catalysts with alternatively connected FePC moieties. The two‐dimensional planar structures could render abundant accessible active sites between layers for fast PMS diffusion and reaction, as well as enhanced conjugation between the central Fe site and the peripheral environment (Figure 2a). The molecular self‐assembly process is shown in Figure S5. The x‐ray diffraction (XRD) patterns of FePPC show three distinct diffraction peaks at 2*θ* = 5.7°, 8.0°, and 27.1°, which are well indexed to the (100), (010), and (001) lattice planes of the AA‐stacking model (Figures 2b,c and S6). The enhanced (100) peak intensity compared to simulation results indicates the dominant exposure of the (100) planes. Fourier transform infrared (FT−IR) spectra exhibit a broad band at 1000−1500 cm^−1^, ascribed to the vibrational absorption of isoindole moieties (Figure 2d). Thermalgravimetric analysis (TGA) of FePC (Figures 2e and S7) shows three exothermic stages at 400°C, 600°C, and 750°C with a weight loss of 60% at 900°C, while FePPC only presents one exothermic stage at 750°C with a weight loss of 47%, indicating that FePPC possesses a higher thermal stability and molecular structural robustness than FePC. Scanning electron microscopy (SEM) images (Figures S8 and S9) show that FePC exhibits rod‐like structures while FePPC presents laminar morphologies, coinciding with the two‐dimensional reticular structures of FePPC. This unique layered structure of FePPC finally leads to a slightly larger SSA of 9.7 m^2^/g, compared with 5.5 m^2^/g of FePC, and therefore more accessible active sites between the layers (Figures S10 and S11).

The element proportion from the energy dispersive spectroscopy coincides with the digestion results from the atomic absorption spectroscopy (Figure S12, 8.9 wt% Fe and 4.9 wt% Fe for FePC and FePPC, respectively). The lower iron content in FePPC is attributed to the co‐existence of FePC and 29H,31H‐phthalocyanine (H_2_PC), as shown in Figure 2c. AC‐TEM illustrates that FePPC exhibited high crystallinity, and the lattice space of 1.5 nm corresponded to the (100) plane (Figure 2f,g), consistent with the enhanced (100) plane exposure in XRD results [32]. The evenly distributed element mapping further confirms that FePC and FePPC are composed of single components, and the atom‐overlapping Gaussian‐function‐fitting mapping derived from Figure 1f well conforms to the chessboard structures of FePPC, suggesting the atomic dispersion of iron atoms in the framework (Figures 2h,i and S13).

**FIGURE 3:**
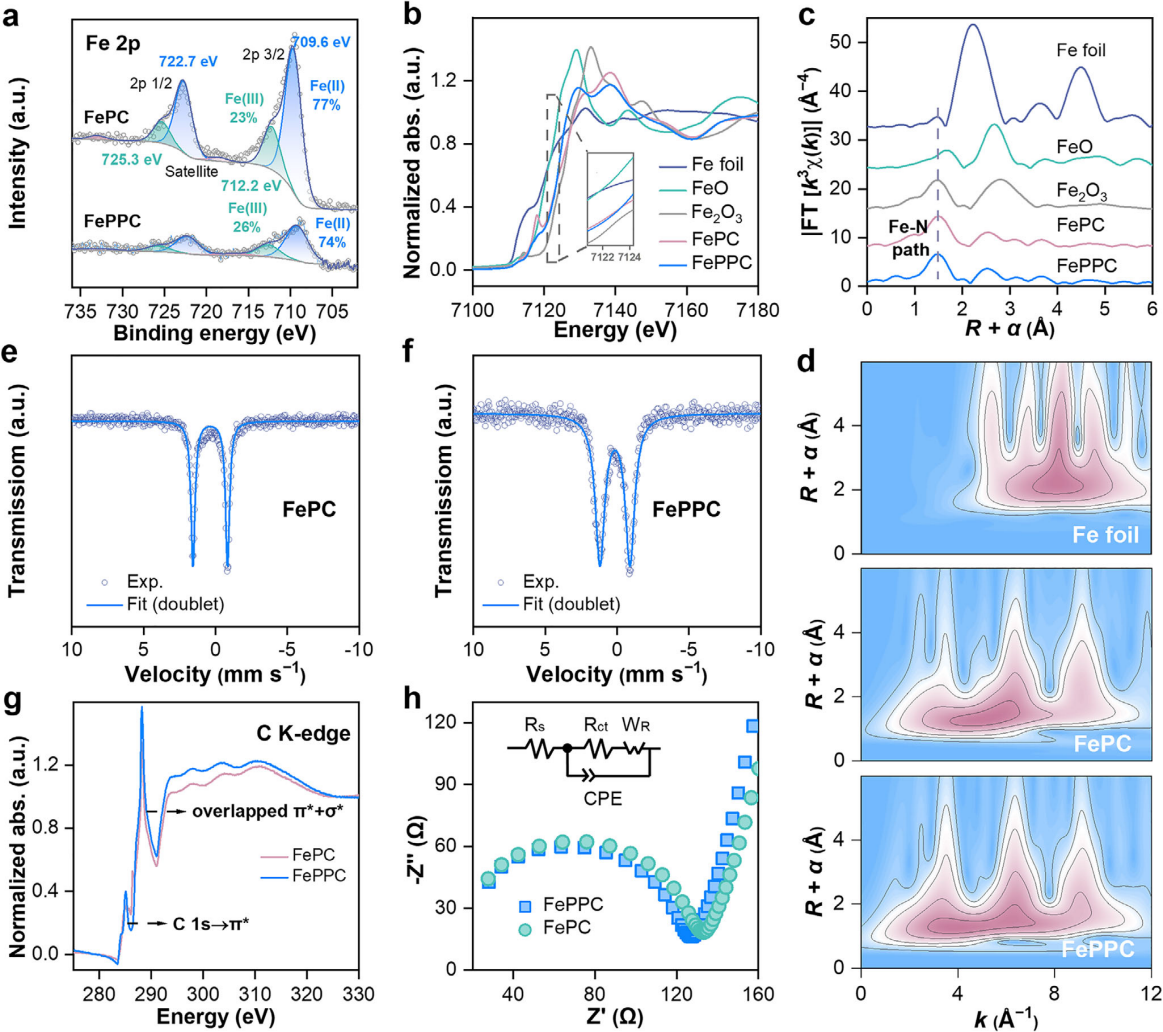
(a) Fe2p XPS spectra of FePC and FePPC. (b) Normalized Fe K‐edge XANES spectra of FePC and FePPC. (c) Fourier‐transformed *k* ^3^‐weighted Fe K‐edge EXAFS spectra. (d) Corresponding *k* ^3^‐weighted Fe K‐edge WT‐EXAFS data. ^57^Fe Mössbauer spectra of (e) FePC and (f) FePPC. (g) C K‐edge NEXAFS spectra of FePC and FePPC. (h) Nyquist plots of FePC and FePPC.

XPS was further conducted to probe the differences in the chemical environments between FePC and FePPC. Fe 2p spectra demonstrate that both FePC and FePPC are composed of a mixture of Fe(II) and Fe(III) states, with Fe(II) accounting for approximately 77% and 74% of the total iron species, respectively (Figure 3a). To further disclose the electronic and coordination structures of Fe atoms, we performed Fe K‐edge analysis. The XANES spectra show that the absorption edge energies of FePC and FePPC are nearly identical and situated between FeO and Fe_2_O_3_ with a valence state of about +2.4 (Figure 3b). Fourier‐transformed EXAFS spectra of FePC and FePPC exhibit the dominant peaks at 1.49 and 2.51 Å, corresponding to the Fe─N path in the first shell and the Fe─C path in the second shell (Figure 3c) [33]. In addition, no peak of Fe−Fe path (2.2 Å for Fe foil, and 2.7 Å for FeO) is observed, indicating that the synthesized FePPC does not contain metallic iron or iron oxide components. Wavelet transforms of FePPC EXAFS results to show a similar pattern at (4.8 Å^−1^, 1.48 Å) as that of FePC (Figure 3d). The EXAFS fitting results also show that FePPC has the same Fe coordination number (4) as FePC (Figure S14 and Table S2). We further utilized ^57^Fe Mössbauer spectra to investigate the electron configurations of central Fe atoms (Figure 3e,f and Table S3). Generally, the structural differences of the active sites can be accurately reflected by changes in isomer shift (IS) and quadrupole splitting (QS) values [34]. Both FePC and FePPC present a primary quadrupole doublet peak with a low IS (< 0.5 mm^−1^) and QS value (< 2.5 mm^−1^), suggesting that the iron atoms in FePC and FePPC exist in the form of intermediate‐spin Fe^2+^ [35]. Notably, the smaller QS value of FePPC can be ascribed to the decreased electric field gradient around Fe on extended conjugated framework.

To further reveal their structural features, we examined their C K‐edge near‐edge x‐ray absorption fine structure (NEXAFS) spectra (Figure 3g). The C K‐edge spectra are primarily composed of two components, including the 1s → π* transition and overlapped π* + σ* transition [36]. Obviously, FePPC exhibits a more pronounced π* peak, indicating a higher degree of π conjugation due to covalent bonding between molecular units. In contrast, the N K‐edge and Fe L‐edge spectra of the two catalysts show similar patterns, consistent with the above discussion of their iron electronic structures (Figure S15). The higher proportion of C−C bond in C 1s XPS spectra also confirms the extended conjugation across the two‐dimensional structure (Figure S16) [37]. Nyquist plots and equivalent circuit fitting reveal that FePPC exhibits a lower charge‐transfer resistance of 120 Ω compared with 128.3 Ω for FePC, indicating enhanced electron transport within the conjugated framework (Figure 3h and Table S4). Moreover, the stronger ultraviolet‐visible absorption ability also corroborates the extended conjugation of FePPC (Figure S17).

To reveal the effects of increased polymerization on energy levels, we conducted quantum chemical calculations of isolated FePC molecules connected by different numbers of monomer units, denoted 2FePC, 3FePC, 4FePC, and 5FePC (Figure S18). The increase of conjugation degrees had a negligible impact on the electron configurations of the Fe sites, with *S* = 1 (intermediate spin state) remaining the ground state for all FePC molecules. Furthermore, the highest occupied molecular orbital (HOMO) and lowest unoccupied molecular orbital (LUMO) energy levels are displayed in Figure S19. As the conjugation degree increases, the HOMO–LUMO gap progressively decreases from 2.62 eV for FePC to 1.56 eV for 5FePC, which promotes electron delocalization from the Fe site toward the conjugated framework, facilitates the intramolecular electron transfer, and subsequently regulates the activity of the catalytic Fe center [30].

### 2.2 Catalytic Performance

**FIGURE 4:**
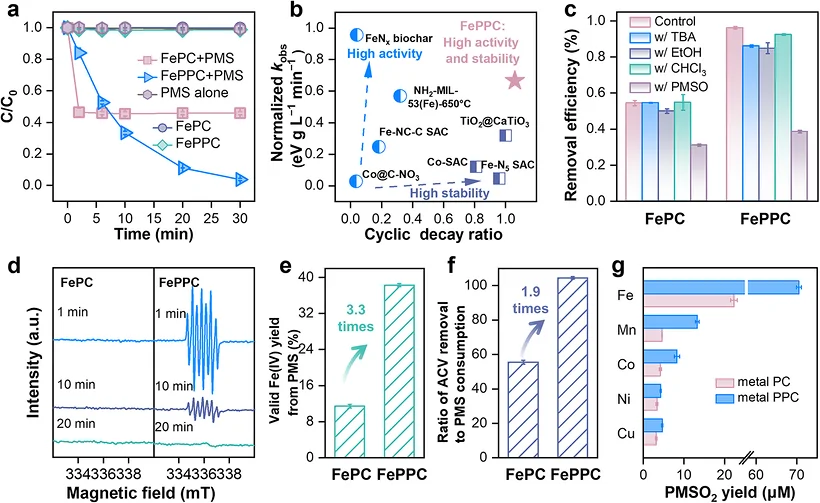
(a) Comparision of the activity of FePC and FePPC for ACV degradation. Experimental conditions: [PMS] = 0.2 mM, [catalysts] = 0.1 g/L, [ACV] = 20 µM. (b) Comparison of the normalized *k*_obs_ and cyclic degradation performance of different catalysts. (c) Quenching experiments for FePC/PMS and FePPC/PMS systems. (d) EPR spectra of FePC/PMS and FePPC/PMS systems using DMPO as a spin trap. Compariosn of (e) Fe(IV) conversion ratio from PMS and (f) Normalized ACV removal efficiency between FePC/PMS and FePPC/PMS systems. (g) Universality of conjugation strategies in stabilizing other high‐valent metal‐oxo species.

We further evaluated the catalytic performance of FePC and FePPC in activating PMS to degrade acyclovir (ACV), a typical antiviral medication. Figure 4a illustrates that both the adsorptive removal of ACV by FePC and FePPC and its direct oxidation by PMS are negligible. In the presence of PMS, FePC exhibited two‐stage kinetics, with 54% ACV removal in the first 2 min and negligible removal thereafter, possibly due to rapid PMS consumption. In contrast, FePPC/PMS gradually degraded ACV, achieving 97% removal in 30 min, demonstrating sustained PMS activation by FePPC for ACV oxidation. Consistently, PMS was quickly depleted within 2 min in the FePC/PMS system but was gradually consumed in the FePPC/PMS system, implying that PMS was predominantly consumed by self‐quenching reactions in FePC/PMS rather than being effectively involved in ACV degradation (Figure S20).

We further traced the decomposition products of PMS to evaluate its utilization efficiency (Figure S21). In the absence of ACV, FePC showed a faster initial O_2_ evolution rate, yielding 92 µmol O_2_ in 30 min, while FePPC produced O_2_ at a much slower rate. In particular, the presence of ACV significantly inhibited O_2_ evolution in FePPC/PMS, thereby markedly improving PMS utilization efficiency.

Considering that the different morphologies and iron content might influence the accessible active site amount, we further altered the substrate dosage to investigate whether such factors played a dominant role in affecting such two‐stage and one‐stage kinetic differences. Increasing the initial catalyst and PMS dosage, or decreasing the ACV concentration, accelerated ACV degradation rates in both systems but did not alter the two‐stage kinetics for FePC. Even if the iron content of FePC is 1.82 times higher than that of FePPC, the final ACV removal efficiency did not greatly increase upon reducing the FePC dosage, suggesting that the intrinsic site reactivity contributed more to the two‐stage kinetics (Figure S22). Moreover, acidic conditions facilitated ACV degradation in both FePC/PMS and FePPC/PMS systems, as the elevated zeta potential of the catalyst surface enhanced electrostatic attraction toward PMS (Figures S23 and S24). In summary, despite the faster ACV degradation rate initially in the FePC/PMS system (ascribed to the higher iron content and stronger redox capacity, which would be further discussed later), most of the oxidant and ROS were wastefully consumed during the process, leading to much lower overall ACV removal efficiency and PMS utilization efficiency.

We further compared the cyclic performances of FePC and FePPC (Figure S25). During five consecutive runs, FePPC maintained its high removal efficiency while the activity of FePC gradually deteriorated. Comprehensive characterizations of the used FePPC catalysts using FTIR, SEM, XRD, XANES, XPS, and N_2_ adsorption–desorption isotherms suggested that FePPC retained pristine structural and chemical properties after the reaction (Figures S26–S32). Besides, it was clear that FePC/PMS system rapidly released substantial iron (1 mg/L) content due to the excessively strong redox properties, whereas the FePPC/PMS system maintained a low degree of iron leaching over time and during cyclic runs, and the leached iron made an insignificant contribution to ACV degradation (Figures S33 and S34). Overall, FePPC exhibited outstanding performance for ACV degradation with PMS, outperforming most state‐of‐the‐art single‐atom Fe catalysts in both activity and stability (Figure 4b and Table S5).

Subsequently, we analyzed the dominant ROS generated in two systems. Tert‐butanol (TBA), ethanol (EtOH), and CHCl_3_ were selected as the quenchers of ^•^OH, SO_4_^•−^/^•^OH, O_2_^•−^, respectively (Figures 4c and S35) [38, 39]. Clearly, TBA, EtOH and CHCl_3_ showed negligible inhibition on ACV degradation, excluding radical‐driven oxidation. Nitrobenzene (NB) and dimethyl phthalate (DMP) were used as the ^•^OH probe, while benzoic acid (BA) was used to further confirm the contribution of ^•^OH/SO_4_^•−^. The probe results also indicated that neither ^•^OH nor SO_4_^•−^ accounted for pollutant degradation in FePC/PMS and FePPC/PMS systems (Figure S36). Singlet oxygen sensor green (SOSG) was applied to probe the generation of ^1^O_2_; however, no characteristic absorption peak of the SOSG‐^1^O_2_ adduct at 530 nm was observed in UV–vis spectra, in sharp contrast to the benchmark ^1^O_2_‐generating HOCl/H_2_O_2_ system (Figure S37). Peroxide‐catalyst premixing experiments were conducted to probe the possible contribution from the electron‐transfer oxidation regime [40]. After premixing the catalysts with PMS for 1, 4, 10, or 30 min prior to pollutant addition, ACV oxidation in both FePC/PMS and FePPC/PMS systems progressively declined with extended premixing time, indicating that the reactive species would decompose in the absence of contaminants and thereby excluding the electron‐transfer oxidation regime (Figure S38). To discern the active sites on FePC and FePPC, we used KSCN as a selective metal‐chelating agent to probe the contribution of Fe sites [41]. As the increase in KSCN concentration from 10 to 100 µM, ACV removal was nearly completely suppressed (Figure S39). Besides, metal‐free H_2_PC exhibited negligible PMS activation activity (Figure S40). These results confirmed that single Fe sites in FePC and FePPC serve as the dominant active sites for PMS activation and ROS generation.

Methyl phenyl sulfoxide (PMSO) was used as a selective probe for high‐valent Fe(IV) species; its addition greatly suppressed the ACV removal efficiency by 23% and 60% in the FePC/PMS and FePPC/PMS systems, respectively, suggesting the generation of Fe(IV) serving as primary oxidizing species for ACV oxidation (Figure S35) [19]. To quantify the proportion of Fe(IV), we assessed the yield of methyl phenyl sulfone (PMSO_2_) from PMSO (Figure S41) [42]. Both FePC/PMS and FePPC/PMS maintained 100% PMSO_2_ yield throughout the reaction, indicating the high selectivity of FePC and FePPC for PMS activation toward selective Fe(IV) generation. The electron paramagnetic resonance (EPR) signals of two systems were also recorded using 5,5‐dimethyl‐1‐pyrroline N‐oxide (DMPO) as a spin trap (Figure 4d). FePPC presented prominent peaks of 5,5‐dimethyl‐2‐oxopyrroline‐1‐oxyl (DMPOX) at the first 1 min due to the over‐oxidation of DMPO or DMPO‐OH adduct by Fe(IV) via hydrolysis and ketonization reactions [17, 43], while the signal gradually diminished due to further decomposition of DMPOX by Fe(IV). In contrast, FePC/PMS manifest silent EPR signals from 1 to 30 min, which well corresponded to its fast Fe(IV) generation and decomposition kinetics.

Overall, FePPC achieved a 3.3‐fold higher Fe(IV) production rate, leading to a 1.9‐fold enhancement in ACV removal compared with FePC (Figure 4e,f). Of note, we also compared the valid Fe(IV) yield with varied catalyst and PMS dosage to normalize the effects of accessible active sites induced by different iron contents and specific surface area in FePC and FePPC (Figure S42). Clearly, the valid Fe(IV) yield in the FePPC/PMS system was maintained at about 40% at different initial conditions, while the valid Fe(IV) yield gradually decreased from 12% to 8% in the FePC/PMS system. We also synthesised two additional catalysts, namely FePPC with a low polymerization degree (FePPC‐LP) and FePC supported on carbon nanotubes (FePC@CNT) to investigate the universality of such strategy. XRD results (Figure S43) suggest that the FePPC‐LP exhibited no characterization peaks at low angles due to the low polymerisation degree achieved using FeCl_3_ as the iron source. Besides, FePC@CNT exhibited no characterization peaks of FePC, indicating FePC was homogeneously dispersed on CNT surface. Similar to FePPC, these two catalysts with enhanced in‐plane conjugation degrees promoted PMSO_2_ generation and suppressed PMS consumption, with a much improved valid Fe(IV) yield from PMS compared with FePC. Notably, both the PMSO_2_ generation amount and PMS utilization efficiency increased in the Mn‐based conjugated system (Figures 4g and S44), which preliminarily suggests that this molecular conjugation‐based stabilization strategy may be extendable to high‐valent manganese oxo species. A full demonstration of this generality across other high‐valent metal‐oxo systems remains to be established.

### 2.3 Fine‐Tuned Fe(IV) Reactivity and Stability

**FIGURE 5:**
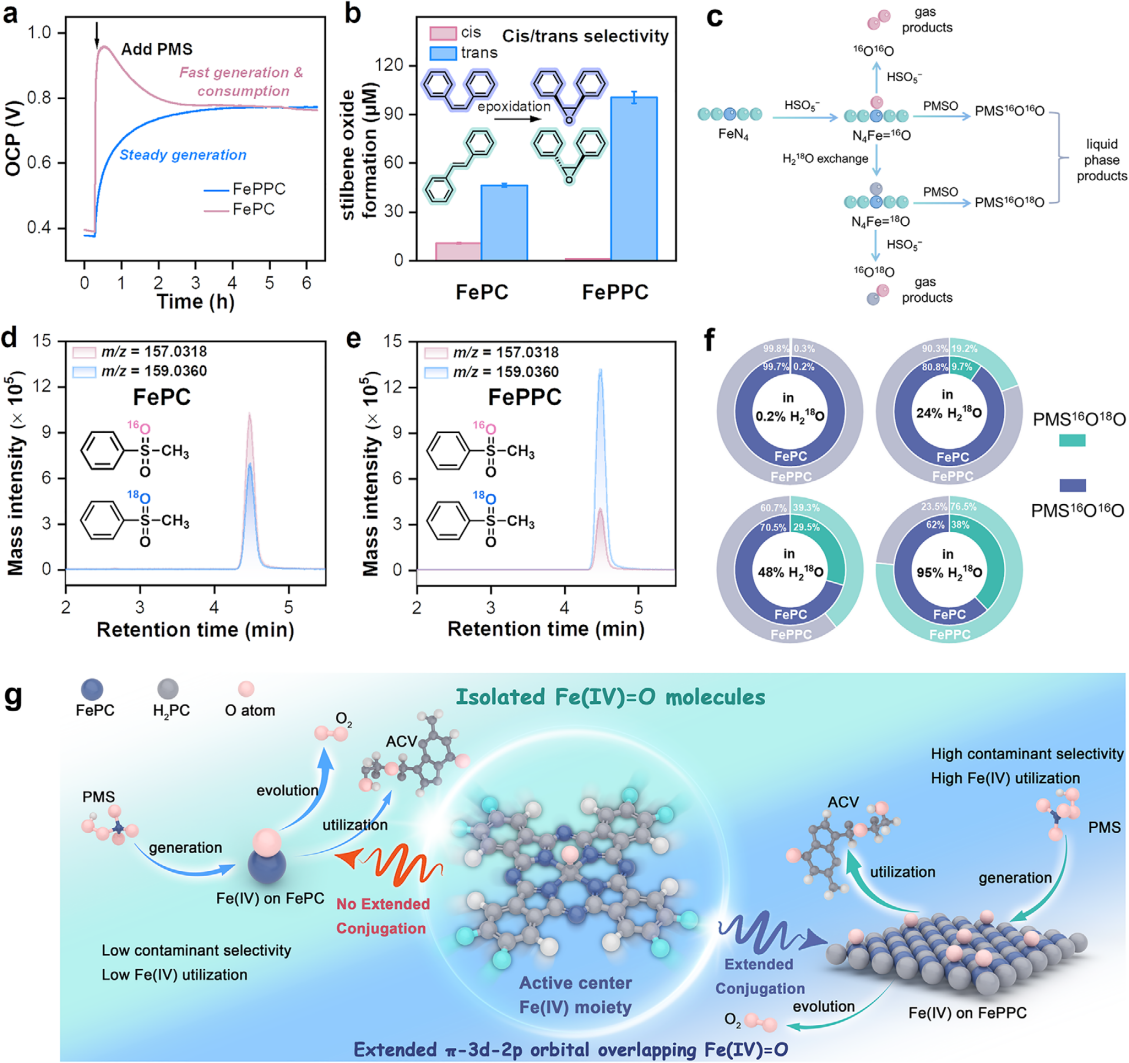
(a) Open circuit potential profiles as a function of time. (b) Comparison of the *cis/trans* selectivities of the Fe(IV) generated in FePC/PMS and FePPC/PMS systems. (c) Schematic illustration of the generation of ^18^O‐labeled PMSO_2_ and O_2_. Extracted ion chromatography of *m/z* = 157.0318 and *m/z* = 159.0360 in (d) FePC/PMS and (e) FePPC/PMS systems. (f) Effects of initial H_2_^18^O concentration on the proportion of isotope‐labelled products. (g) Mechanism illustration of the optimized Fe(IV) reactivity in FePPC/PMS system toward more organic contaminant degradation and less O_2_ evolution.

The redox capacity of Fe(IV) was measured using electrochemical approaches to assess its reactivity and stability [44]. As depicted in Figure 5a, the open circuit potential of FePC after PMS addition quickly climbed to 0.96 V and then decreased to 0.78 V upon reaching the plateau. Further increasing the PMS dosage elevated the peak potential but also shifted the inflection point, indicating faster Fe(IV) generation, which was then rapidly consumed by PMS (Figure S45). In comparison, the potential of FePPC gradually increased to 0.78 V, without a significant decline even at higher PMS dosages, indicating the exceptional stability of Fe(IV) in FePPC.

We then used *cis*‐stilbene and *trans*‐stilbene as featured probes to compare the reactivity and stability of Fe(IV) generated on FePC and FePPC. Fe(IV) epoxidizes *cis*‐stilbene and *trans*‐stilbene into *cis*‐stilbene oxide and *trans*‐stilbene oxide, respectively, via oxygen transfer reaction; however, the energy barrier for the epoxidation of *cis*‐stilbene is higher than that of *trans*‐stilbene, resulting in a low selectivity toward *cis*‐stilbene oxide (Figure S46). Such energy barrier could be overcome, if the reactivity of Fe(IV) species is high enough, leading to greater selectivity toward *cis*‐stilbene oxide when reacting with a mixture of *cis*‐ and *trans*‐stilbene. Nam et al. [45] also found that Fe(IV) with axial Cl coordination exhibited higher reactivity and reacted faster with *cis*‐stilbene to generate *cis*‐stilbene oxide, compared with Fe(IV) with axial CH_3_CN coordination. Herein, we conducted competitive kinetic experiments using excess *cis*‐ and *trans*‐stilbene to compare the selectivity of Fe(IV) species in two catalysts toward corresponding *cis*‐ and *trans*‐stilbene oxides (Figures 5b and S47).

Clearly, FePC/PMS and FePPC/PMS systems preferentially reacted with *trans*‐stilbene to produce trans‐stilbene oxide. However, the FePC/PMS system still generated 10.9 µM cis‐stilbene oxide with a *cis*‐trans selectivity of 1:4.27, while negligible cis‐stilbene oxide was generated in the FePPC/PMS system. Notably, neither *cis*‐stilbene oxide nor trans‐stilbene oxide was further degraded in FePC/PMS or FePPC/PMS (Figure S48), excluding the possibility that cis‐stilbene oxide was generated but further decomposed in the FePPC/PMS system. Therefore, Fe(IV) generated on FePC showed a higher oxidative capacity than Fe(IV) generated on FePPC, consistent with the electrochemical analyses in Figure 5a, contributing to its high reactivity with PMS.

We further conducted in‐situ Raman spectra measurement to probe the formation of Fe(IV) on FePC and FePPC. As shown in Figure S49, a stronger peak at 831 cm^−1^ could be observed in FePPC/PMS system compared with FePC/PMS system, suggesting the slow decomposition kinetics of Fe(IV) on FePPC [15]. Kinetic isotope effect (KIE) studies showed that FePC exhibited a smaller normalized KIE than FePPC (Figure S50). Normally, a smaller KIE value in the FePC/PMS system suggests a weaker inhibition effect of N─H bond deuteration in D_2_O, indicating that the FePC‐generated Fe(IV) species with higher reactivity prefer to proceed via proton‐coupled electron transfer reaction; this effect further leads to rapid PMS depletion toward O_2_ evolution, bypassing the on‐demand pathway to supplying oxidative Fe(IV) species and prohibiting pollutant degradation [45, 46].

We further employed ^18^O isotope labelling to trace the incorporation of oxygen into both aqueous and gaseous intermediates. Due to the reversible hydration‐dehydration reaction [47, 48, 49], Fe(IV)═O generated from surface Fe(II) via PMS activation is partially labeled with ^18^O when reacting in H_2_^18^O solvent, and then oxidizes PMSO through oxygen transfer reaction and eventually produces both PMS^16^O^16^O (*m/z* = 157.0318) and PMS^16^O^18^O (*m/z* = 159.0360) (Figure 5c)_._ Notably, the proportion of PMS^16^O^18^O was substantially higher for FePPC (76.5%) than for FePC (38%) in 95% H_2_^18^O (Figure 5d,e). Upon reducing the H_2_^18^O proportion in the reaction medium to 48%, 24%, and 0.2%, the proportions of PMS^16^O^18^O in the FePPC/PMS system consistently remained higher than those in the FePC/PMS system (Figure 5f). The higher ^18^O proportion of PMSO_2_ in the FePPC/PMS system indicated more oxygen exchange between Fe(IV) and the solvent prior to PMSO attack, implying the moderate redox potential and enhanced stability of Fe(IV) on FePPC. Additionally, the analysis of gaseous products shows a higher ^18^O abundance in O_2_ evolved from the FePPC/PMS system, which is consistent with the increased ^18^O incorporation observed in the generated Fe(IV) (Figure S51).

Overall, the fates of Fe(IV) in FePC/PMS and FePPC/PMS systems were illustrated in Figure 5g. Both FePC and FePPC can activate PMS to produce surface Fe(IV) species. The extended conjugation network stabilizes Fe(IV) on FePPC, providing sufficient oxidative capacity to attack contaminants and preventing its further reaction with PMS for O_2_ generation. As a result, FePPC/PMS effectively leverages the generated Fe(IV) redox for selective ACV removal, achieving high Fe(IV) and PMS utilization efficiencies.

### 2.4 Theoretical Insights Into the Customization of Fe(IV) Reactivity

**FIGURE 6:**
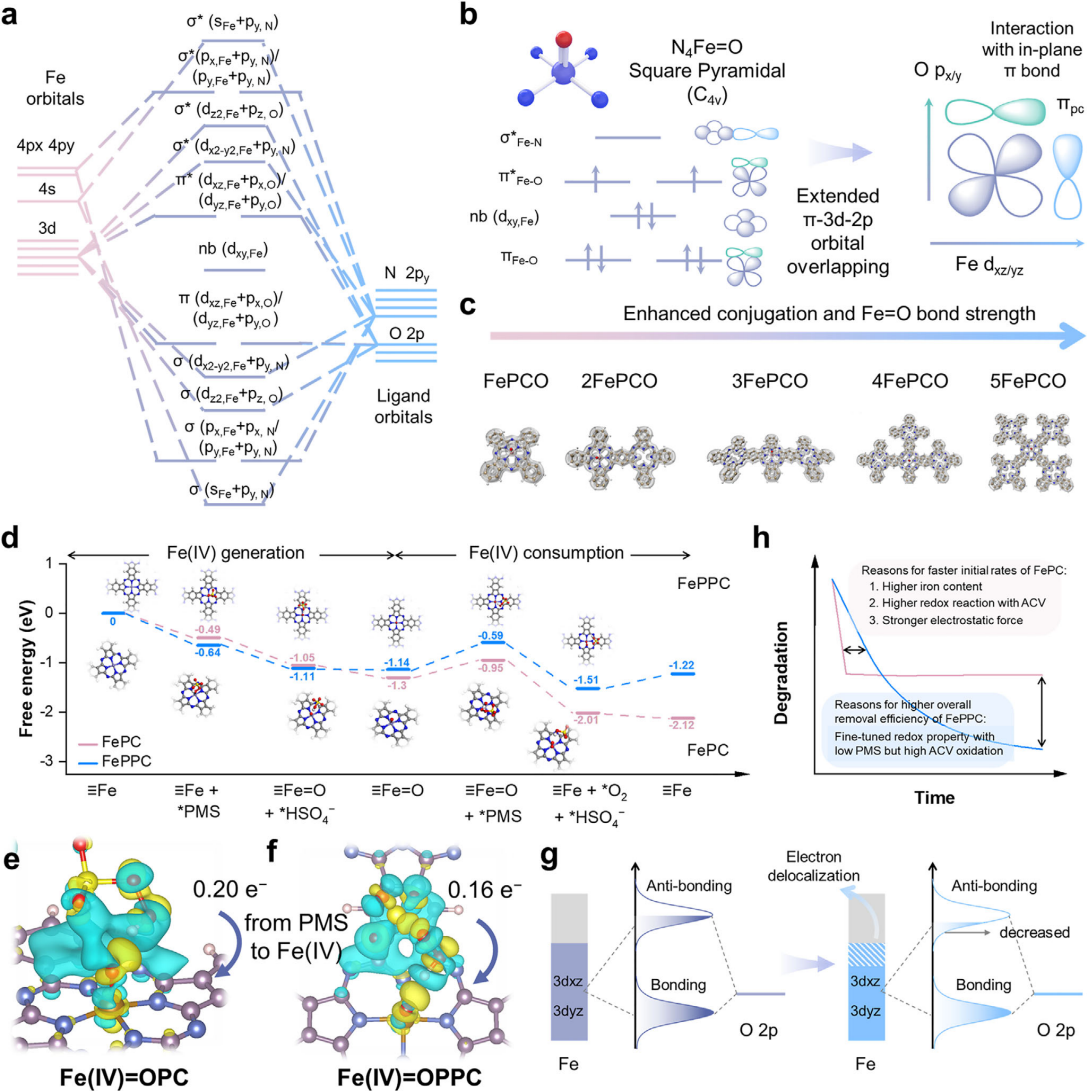
(a) Molecular orbital energy levels of N_4_Fe═O structures with C_4v_ symmetry. (b) Frontier orbital electron distribution and illustration of the interaction between π, Fe_3d_, and O_2p_ orbitals. (c) Localized orbital locator analysis of the π electron cloud distribution of isolated FePC molecules with different conjugation degrees (d) Gibbs free energy change along with the generation of Fe(IV)═O and consumption of Fe(IV)═O for O_2_ evolution. The charge difference distribution of PMS adsorption models on (e) Fe(IV)═OPC and (f) Fe(IV)═OPPC. The blue area around PMS molecules indicated the electron depletion in PMS. (g) Illustration of the conjugation‐induced electron delocalization effects on Fe(IV)═O bond composition. (h) Illustration of the reasons for initial reaction rate differences and overall removal efficiency differences.

We further conducted theoretical analysis to gain insights into the origins of distinct reactivity and stability of Fe(IV). Based on group theory, the N4Fe═O moiety belongs to C4v symmetry, with molecular orbital energy levels as shown in Figure 6a,b. The frontier molecular orbitals are mainly composed of π(Fe−O) < non‐bonding Fe 3dxy < π*(Fe−O) < σ*(Fe−N) with two unpaired electrons distributed on degenerate π*(d_xz,Fe_ + d_x,O_) and π*(d_yz,Fe_ + d_y,O_) orbitals [50]. When the N_4_Fe═O moiety is generated on the FePC molecule (named FePCO), the peripheral ligand N atoms form delocalized in‐plane π bonds with four isoindole rings, which further overlap with both π bonds of the Fe(IV)═O moiety (consisting of Fe 3d and O 2p orbitals). With the increase of polymerization degrees (2FePCO, 3FePCO, 4FePCO, and 5FePCO manifest that the conjugation units increased from 2 to 5), the π‐Fe3d–O2p overlapping is further enhanced across the two‐dimensional molecular framework. As shown in Figure 6c, the localized orbital locator analysis of π orbitals indicated that π electrons spread across the FePCO molecules, giving rise to enlarged electron‐conjugated regions with the increased isoindole units.

To reveal the composition and interaction of molecular orbitals, we further conducted second‐order canonical perturbation analysis (SCPA) for the frontier orbitals containing π*(Fe─O) components (Figure S52). The proportion of O 2p_x_/2p_y_ and Fe d_xz_/_yz_ in different molecular orbitals of FePCO (from 50% to 1%) was much higher than that in 5FePCO (all less than 1%), suggesting that the increased conjugated units enhanced the interaction between the Fe(IV) = O moiety and the lateral π bonds. As a result, the electrons in the anti‐bonding π*(Fe─O) orbitals are delocalized across the macromolecules, verified by the decreased electron density at the bond critical point of Fe(IV)═O bond (Figure S53a). In addition, the increase in Mayer bond order from FePCO to 5FePCO also indicated that the electron occupancy is depleted at the anti‐bonding π*(Fe−O) orbitals (Figure S53b). As a result, the Fe(IV)═O bond was greatly strengthened with the bond energy increasing from 58.8 to 72.2 kcal/mol (Figure S54), which may prohibit the dissociation via reacting with PMS.

We further conducted first‐principles calculations to investigate the free energy change from Fe(IV) generation to Fe(IV) consumption on FePC (denoted as Fe(IV)═OPC) and FePPC (denoted as Fe(IV)═OPPC, Figure 6d). As shown in Figure S55, the adsorption and the cleavage of PMS peroxy bond to form Fe(IV) are both thermodynamically favorable for FePC and FePPC, with the terminal O atom (near H atom) adsorbed on the Fe site being the most favorable configuration. As a result, FePPC exhibited greater Gibbs free energy change (‐0.64 eV than ‐0.49 eV), which should be ascribed to the stronger covalent interaction between Fe atom and O_PMS_ atom to form a more robust Fe(IV)═O bond. However, all the adsorption configurations of PMS on Fe(IV)═OPC and Fe(IV)═OPPC were endothermic and the configuration involving hydrogen bonding between the terminal H atom of PMS and the O atom of Fe(IV)═O is the most energetically favorable, implying the proton‐coupled electron transfer pathway facilitates further PMS decomposition over Fe(IV)═O (Figure S56). We further conducted a charge‐difference analysis to reveal electron transfer between PMS and the Fe(IV)═ O moiety (Figure 6e,f). Bader charge analysis indicated that PMS donated 0.2 and 0.16 electron to Fe(IV)═OPC and Fe(IV)═OPPC, respectively. Accordingly, the energy barrier for Fe(IV) decomposition on Fe(IV)═OPC was 0.2 eV lower than that on Fe(IV)═OPPC, suggesting Fe(IV)═OPPC with optimized reactivity was more stable against the attack of PMS.

The bonding and anti‐bonding orbital filling was demonstrated in Figures S57 and 6g. The crystal orbital Hamilton populations (COHP) analysis for the Fe(IV)═O bond revealed that the electron population below the Fermi level in Fe(IV) = OPC contains more anti‐bonding components, and the integration of COHP below the Fermi level (1.41) was also smaller than Fe(IV) = OPPC (1.61), suggesting the more robust Fe(IV)═O bond in Fe(IV)═OPPC [51, 52]. The enhanced delocalization of electrons in Fe(IV)═O π* anti‐bonding orbital would promote the formation of Fe(IV)═O bond between the Fe site and PMS molecules due to increased covalency, while decreasing the tendency of Fe(IV)═O moiety to abstract electrons from PMS because of the inhibited electron pairing tendency.

Finally, we elucidated the distinct degradation performances of FePC/PMS and FePPC/PMS systems in Figure 6h. Owing to the higher iron content, higher redox potential with ACV, and stronger surface electrostatic interaction, FePC exhibited faster initial degradation kinetics compared with FePPC. However, the more reactive and less selective nature of Fe(IV)═O in FePC/PMS results in significant Fe(IV) decomposition via further reaction with PMS, thereby decreasing overall pollutant removal efficiency.

### 2.5 Practical Application

**FIGURE 7:**
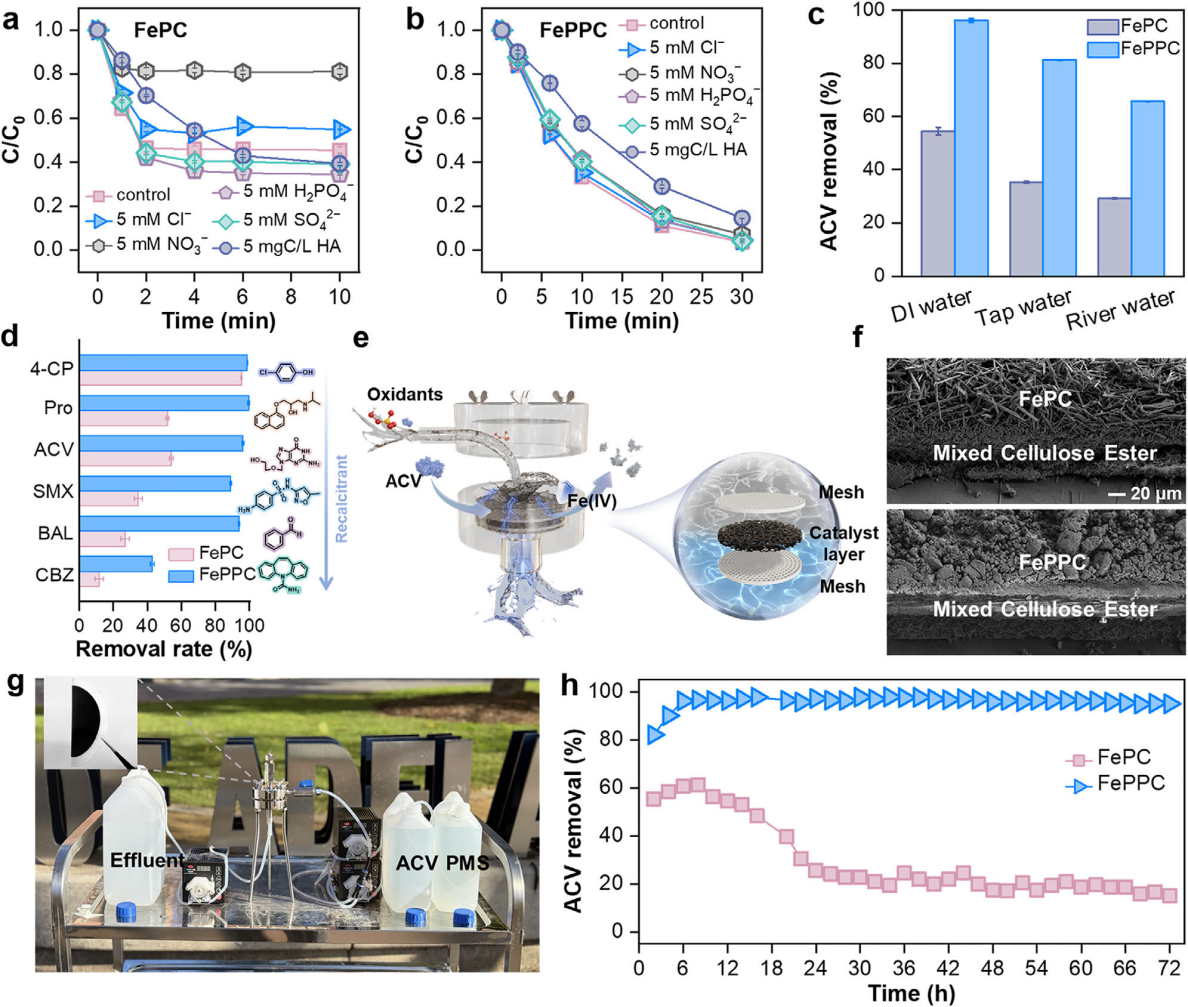
Effects of common water matrices on the degradation performance of (a) FePC/PMS and (b) FePPC/PMS systems. (c) Evaluation of the degradation performance of the FePPC/PMS system in different sources of water. (d) Comparison of the performance of FePC/PMS and FePPC/PMS systems toward contaminants with different structures. (e) Demonstration of the membrane filtration apparatus for continuous catalytic wastewater treatment process. (f) Composition of catalyst layer membrane. (g) Pilot‐scale demonstration of a membrane‐based flow‐through apparatus. (h) Long‐term ACV removal performance of FePC/PMS and FePPC/PMS systems.

The performances of FePC and FePPC in the presence of diverse water backgrounds containing anions and organic matter were assessed. Figure 7a,b shows that Cl^−^, NO_3_^−^, and humic acid significantly influenced the removal efficiency of FePC, possibly ascribed to their adsorptive screening and scavenging of the highly active Fe(IV) species on FePC. In contrast, FePPC exhibited strong tolerance to Cl^−^, NO_3_^−^, H_2_PO_4_^−^, SO_4_^2−^, while humic acid induced only a minor decline in removal efficiency. When the reaction medium was exchanged to tap water or river water (Torrens River, Adelaide), FePPC retained high selectivity toward ACV removal, indicating its strong potential for selective contaminant removal under realistic water conditions (Figures 7c and S58). Also, FePPC outperformed FePC in the effective degradation of diverse contaminants, including 4‐chlorophenol, sulfamethoxazole, carbamazepine, benzaldehyde, and propranolol (Figure 7d), demonstrating that the stabilization strategy preserved sufficient Fe(IV) reactivity for attacking organic contaminants. Analysis of the degradation products of ACV demonstrates that Fe(IV) oxidises ACV through the N‐dealkylation on imidazole N atom to form TP151 ([M+H]^+^ = 152.0570) and ring‐open reactions to form TP229 ([M+H]^+^ = 230.0882) and TP213 ([M+H]^+^ = 214.0946), all of which would finally transform into small molecule TP113 ([M+H]^+^ = 114.0923) (Figure S59 and Tables S7)

We further established a catalytic membrane system for flow‐through wastewater treatment (Figure 7e–g) [53, 54]. ACV and PMS were continuously pumped into the membrane reactor with FePC/FePPC as the active layers and the mixed cellulose as the substrates. As demonstrated in Figure 7h, FePPC maintained a high ACV removal efficiency during 72 h treatment period with above 95% ACV removal and a normalized treatment efficiency of 66.76 L·eV·day^−1^·g^−1^. However, the removal performance of FePC quickly reached a plateau of 60% and then dropped to below 20%, suggesting the higher long‐term operational stability of FePPC. In addition, the iron leaching from FePPC was far below that from FePC throughout the process, and also below the National Secondary Drinking Water Regulations of the US Environmental Protection Agency (0.3 mg/L; Figure S60). Overall, the results highlight the great potential of FePPC for chemical‐effective and high‐performance water treatment.

## 3 Conclusion

The atom utilization efficiency of oxidants and ROS represents a critical yet often overlooked limitation in AOP‐based wastewater treatment technologies. Compared with conventional radical oxidation, the tunable reactivity of Fe(IV) endows this pathway with greater flexibility across diverse application scenarios while enabling optimized reaction kinetics. In this work, we pioneered an in‐plane conjugation strategy to precisely regulate Fe(IV) reactivity, thereby suppressing its side reactions with PMS and enhancing its utilization efficiency for pollutant oxidation. The two‐dimensional reticular FePPC framework consisting of alternatively connected FePC sub‐structures not only provided substantial accessible active sites within the layered structures, but also extended the in‐plane conjugation, which could strengthen π–Fe 3d–O 2p orbital overlap and promote electron delocalization along the Fe(IV)═O bond, and lead to reduced occupancy of anti‐bonding π* orbitals. This electronic modulation reinforces the Fe(IV)═O bond, tuning redox reactivity and selectivity toward contaminant oxidation. As a result, the system achieves a 3.2‐fold increase in Fe(IV) utilization efficiency. Overall, this conjugation‐enabled molecular engineering strategy addresses an important knowledge gap in regulating Fe(IV) redox properties and overcomes key atom‐efficiency bottlenecks in AOPs, thereby offering a viable pathway toward more selective and chemically efficient water decontamination.

## Author Contributions

**Hongyu Zhou**: conceptualization, data curation, formal analysis, validation, investigation, and writing – original draft. **Junwen Chen**: investigation. **Jiuyi Wang**: investigation, formal analysis. **Xinhao Wang**: investigation. **Qiming Zhang**: formal analysis. **Shiying Ren**: investigation. **Xuning Li**: investigation, formal analysis. **Li Gao**: investigation, writing – review and editing. **Shaobin Wang**: writing – review and editing, supervision, and resources. **Xiaoguang Duan**: conceptualization, formal analysis, funding acquisition, project administration, resources, writing – review and editing.

## Conflicts of Interest

The authors declare no conflicts of interest.

## Supporting information




**Supporting File**: anie73623‐sup‐0001‐SuppMat.docx.

## Data Availability

The data that support the findings of this study are available from the corresponding author upon reasonable request.
