# Supplementary material for: Tailoring High‐Valent Iron‐Oxo Species Redox via Extended π‐Fe3d–O2p Orbital Overlapping Toward Chemical‐Efficient Water Purification
Source: Angew Chem Int Ed Engl. 2026 Jul 13;65(38):e5511823. doi: 10.1002/anie.5511823 (PMC13573064; doi:10.1002/anie.5511823)
Supplement: Supplementary file 1 [file ANIE-65-e5511823-s001.docx]

*A Supporting information for*

**Tailoring High-Valent Iron-Oxo Species Redox via Extended π-Fe3d-O2p Orbital Overlapping towards Chemical-Efficient Water Purification**

Hongyu Zhou^1^, Junwen Chen^1^, Jiuyi Wang^2^, Xinhao Wang^1^, Qiming Zhang^1^, Shiying Ren^1^, Xuning Li^2^, Li Gao^3^, Shaobin Wang^1^*, and Xiaoguang Duan^1^*

^1^ School of Chemical Engineering, Adelaide University, Adelaide, SA 5005, Australia

^2^ State Key Laboratory of Catalysis, Dalian Institute of Chemical Physics, Chinese Academy of Sciences, Dalian 116023, P.R. China

^3^ School of Science, STEM College, RMIT University, Melbourne, VIC 3000, Australia

*Corresponding author: Shaobin Wang, Xiaoguang Duan

Email address: [shaobin.wang@adelaide.edu.au](mailto:shaobin.wang@adelaide.edu.au); [xiaoguang.duan@adelaide.edu.au](mailto:xiaoguang.duan@adelaide.edu.au)

This file contains 11 texts, 60 figures and 7 tables.

**Text S1 Chemicals**

Peroxymonosulfate, acyclovior (United States Pharmacopeia Reference Standard), sulfamethoxazole (SMX, United States Pharmacopeia Reference Standard), carbamazepine (United States Pharmacopeia Reference Standard), benzaldehyde (≥ 99%), 1,2,4,5-tetracyanobenzene (97%), tert-butanol (TBA, anhydrous, ≥ 99.5%), chloroform (99.5%), methyl phenyl sulfoxide (97%),methyl phenyl sulfone (≥ 98% GC), 5,5-dimethyl-1-pyrroline N-oxide (DMPO), potassium perroxydisulfate (ACS reagent, ≥99.0%), benzoic acid (ACS reagent grade, ≥ 99.5%, crystalline), nitrobenzene (ACS reagent, ≥99.0%), potassium thiocyanate (ACS reagent, ≥ 99.0%), water-^18^O (98 atom% ^18^O), deterium oxide (99.9%), sodium chloride (ACS reagent, ≥ 99.0%), sodium phosphate monobasic (ReagentPlus®, ≥ 99.0%), sodium sulfate (ReagentPlus®, ≥ 99.0%), sodium nitrate (ReagentPlus®, ≥ 99.0%), humic acid, iron phthalocyanine and 29H,31H-phthalocyanine were purchased from Sigma Aldrich. Singlet oxygen sensor green (SOSG) was purchased from Thermo. Sulfuric acid (98%), hydrogen peroxide (30 wt%), and ethanol (98%) were purchased from Chem-supply. Propranolol (98%), cis-stilbene, trans-stilbene, cis-stilbene oxide, and trans-stilbene oxide were obtained from Aladdin Industrial Inc. (Shanghai). Iron powder (99.99%, metals basis, 50nm) was purchased from Macklin. Commercial carbon nanotube (OD: 10-30 nm, length: 10-20 μm, purity: ≥ 98%) was obtained from Times Nano, Chengdu Organic Chemicals Co. Ltd. Deionized water was used in all the experiments.

**Text S2 Catalyst synthesis**

**FePPC:** First, 200 mg 1,2,4,5-tetracyanobenzene was thoroughly mixed with 126 mg of iron powder, then ground and transferred to a Pyrex tube. The Pyrex tube was then evacuated and flame-sealed, with a total length of ca. 5.0 cm. The tube was then heated to 400 °C at 5 °C/min and kept for 4 h in a furnace. After cooling to room temperature, the product was washed with 1 M H_2_SO_4_ at 70 °C for 4 h and then rinsed with plenty of water and ethanol. After drying at 60 °C in a vacuum for 24 h, the powder was collected and named FePPC.

**Low-polymerization degree FePPC:** The synthesis procedure is similar to that of FePPC except that 126 mg iron powder was replaced with 365 mg anhydrous FeCl_3_.

**FePC@CNT:** CNT-supported FePC (FePC@CNT) was synthesized using a modified method.^1^ Briefly, 100 mg of commercial CNT was dispersed in 100 mL N,N-dimethylformamide (DMF), forming a uniform solution under ultrasonication for 1 h. Then 10 mg of FePC was added to the above solution with ultrasonic treatment for another 30 min. After that, the mixture was stirred for 48 h at room temperature. The precipitate was then collected by vacuum filtration and sufficiently washed with DMF and ethanol until it was colorless. Finally, the precipitate was lyophilized to yield FePC@CNT.

**Text S3 Material characterisations**

The morphologies of FePC and FePPC were investigated using FE-SEM (SU7000 Hitachi, Japan). AC-TEM images and elemental mapping were conducted on the FEI Titan Themis 80–200. The atom-overlapping Gaussian-function-fitting mapping image was plotted using DigitalMicrograph and Origin software. The original image was first smoothed using fast Fourier transform (FFT) in DigitalMicrograph software. Then the FFT image was filtered using the mask function and the smooth edge was set as 5 pixels. After an inverse FFT process, the modified image was saved as txt file, which would be then used in Origin for 3D plotting. XRD patterns were acquired using an X-ray diffractometer with Cu Kα radiation at a scan rate of 5 °/min (Rigaku MiniFlex 600). *Ex-situ* FT-IR was conducted on a Nicolet iS50 (ThermoFisher, USA). The specific surface areas were measured using the Brunauer-Emmett-Teller (BET) method on a surface area analyzer (V-Sorb 2800P, Ultrametrics). Raman spectra were recorded using a HORIBA LabRAM HR Evolution spectrometer with a 532 nm excitation laser. The surface chemical oxidation states were recorded by X-ray photoelectron spectroscopy and calibrated using the C 1s peak position at 284.8 eV (XPS, AXIS Ultra DLD, Kratos Co., UK). EPR signals were recorded using DMPO as a spin-trapping reagent on a Bruker ESR-5000 spectrometer. Zeta potentials were measured using a Zetasizer Nano-ZS (Malvern, UK). Powder size was analyzed using a Mastersizer 2000 (Malvern, UK). Thermogravimetric analysis was conducted using a TGA/DSC3+ (Mettler Toledo, Switzerland). Mössbauer spectroscopy was measured using Iron Analytics with a ^57^Co γ-ray source and a transmission detector, and the isomer shift values were referenced to that of α iron (Czech Republic). X-ray absorption spectroscopy (transmission mode) and soft X-ray absorption spectroscopy were measured at Australian Synchrotron.

**Text S4 Analytic method**

The concentration of target organics was measured using high-performance liquid chromatography (HPLC, LC-40D XR, SHIMADZU, Japan) equipped with a C18 separation column. The detailed HPLC condition is listed in Table S6. Residual PMS concentration was analyzed using the 2,2′-azino-bis(3-ethylbenzothiazoline-6-sulfonic acid) colorimetric method. The leaching Fe concentration was measured using atomic absorption spectroscopy (Shimadzu 6300, Japan). O_2_ measurement was conducted in an air-tight reactor (connected with gas chromatography), which was pre-filled with catalysts and PMS solution but separated from each other at certain distance. The reactor was first purged with N_2_ for one hour to remove the air in the reactor, and then the catalysts was mixed with PMS solution to initiate the reaction. The gas composition in the reactor was analyzed by gas chromatography at pre-determined time. The oxygen evolution amount was analyzed using gas chromatography with a thermal conductivity detector and CP-Molsieve 5A PT column (Agilent 8890, 20m × 530 μm × 50 μm). The inlet temperature was 100 °C, and the heater temperature of TCD was 250 °C. Reference flow was set as 25 ml/min. The proportion of PMS^16^O^18^O and PMS^16^O^16^O was detected by an ultraperformance liquid chromatography-quadrupole time-of-flight premier mass spectrometer (UPLC-QTOF-MS/MS, Agilent 6500, USA), while the proportion of ^16^O^16^O, ^16^O^18^O and ^16^O^16^O was detected by GC-MS/MS TQ8040 (Shimadzu, Japan). The electrochemical measurements were performed on an electrochemical workstation (CHI 760E) with a glassy carbon electrode as the working electrode, a Pt plate electrode as the counter electrode, and a saturated calomel electrode as the reference electrode. Ink for the electrochemical measurement was prepared by adding 5 mg of catalysts into 0.4 mL of isopropanol and 20 μL of a 5% Nafion solution, followed by sonication for 30 min. The working electrode was prepared by loading 10 μL of ink on the glassy carbon electrode.

**Text S5 Evaluation of organic degradation performance**

For most of the degradation experiments, 0.1 g/L materials were first dispersed in 50 mL, 20 μM contaminant solution under ultrasonic irradiation for a short time. To initiate the experiment, PMS stock solutions were subsequently added; samples were taken at the predetermined time, mixed with 20 μL, 0.1 M Na_2_S_2_O_3_ solution and quickly filtered through a 0.22 μm mixed cellulose ester syringe filter disc for the analysis of residual contaminant concentration. All the experiments were conducted at least duplicate (n ≥ 2) with error bars representing the standard deviation. The degradation of ACV was fitted with a pseudo-first-order model (eq. S1), where C_0_ and C_t_ represented the ACV concentrations initially and at t moment, and *k*_app_ represented the apparent reaction kinetic rate constant.

$\text{ln (}\text{C}_{\text{t}}\text{/}\text{C}_{\text{0}}\text{) = }{\text{-}\text{k}}_{\text{app}}\text{ × t}$	(eq. S1)

**Text S6 Details for PMS^16^O^18^O measurement**

Chromatography was performed with an Agilent 1290 Liquid Chromatography on an EclipsePlusC18 RRHD column (1.8 µm, 3.0×50 mm). The column was maintained at 40 °C and eluted with a gradient mobile phase. The gradient mobile phase ratio of A/B was set as: the ratio kept at 90/10 for the first 6 min, then changed linearly from 90/10 to 40/60 in the next 6 min and held for 5 min, followed by a sharp adjustment to 90/10 in 1 min, and kept for 5 min for re-equilibration, where A is 0.1% (v/v) ammonia aqueous solution and B is acetonitrile with a flow rate of 0.30 mL/min. The mass spectra were recorded in a molecular ion scanning mode (m/z 50 to 1200) in positive electrospray ionization (ESI) modes. Mass spectrometry conditions were: gas flow, 8 L/min; gas temp, 325 ^o^C; sheath gas temp, 350 °C; nebulizer, 45 psig; sheath gas flow, 12 L/min; injection volume, 20 µL.

**Text S7 Details for ACV degradation intermediate measurement**

Chromatography was also performed with an Agilent 1290 Liquid Chromatography on an EclipsePlusC18 RRHD column (1.8 µm, 3.0×50 mm). The column was maintained at 40 °C and eluted with a gradient mobile phase. The gradient mobile phase ratio of A/B was set as: the ratio kept at 85/15 for the first 2 min, then changed linearly from 85/15 to 15/85 in the next 6 min and held for 2 min, followed by a adjustment to 85/15 in 4 min, and kept for 5 min for re-equilibration, where A is pure water and B is acetonitrile with a flow rate of 0.12 mL/min. The mass spectra were recorded in a molecular ion scanning mode (m/z 20 to 1700) in positive electrospray ionization (ESI) modes. Mass spectrometry conditions were: gas flow, 8 L/min; gas temp, 325 ^o^C; sheath gas temp, 350 °C; nebulizer, 45 psig; sheath gas flow, 12 L/min; injection volume, 5 µL.

**Text S8 Details for ^16^O^18^O measurement**

To identify products, we employed a Shimadzu GC-MS/MS TQ8040 system operating in an Electron Ionization (EI) mode, equipped with a RESTEK Rxi-5Sil MS column (30 m × 0.25 mm, 0.25 µm). Helium was used as a carrier gas and argon served as a collision-induced dissociation (CID) gas. The column oven was initially set at 26 °C, with an injection temperature of 200 °C. Total and column flows were maintained at 5 mL/min and 1 mL/min, respectively. The ion source and interface temperatures were set at 230 and 250 °C, respectively. The event time was 0.3 s, and the total run time was 24 min. The temperature program for the oven was as follows: held at 26 °C for 0.5 min, ramped to 70 °C at 2 °C/min, held for 1 min, and then returned to the initial temperature. Detection was conducted in a positive ion mode using a Q1 scan. The mass range monitored was m/z = 2–50. All analyses were performed in duplicate. Data processing was carried out using a Shimadzu LabSolutions software, version 4.54.

**Text S9 Details for theoretical calculation**

The quantum chemical calculation of isolated FePC molecules with different polymerization degrees was conducted using Gaussian 16^2^ and analyzed using Multifwn^3^, while the first-principle calculation of periodic systems was conducted using Vienna ab initio simulation package (VASP 6.5.1) software.^4, 5^

For quantum chemical calculation, the geometry optimization and frequencies of different metal complexes were calculated at the PBE0-GD3BJ/TZVP level, while single-point calculations were carried out at the PBE0-GD3BJ/Def2-TZVP level on the optimized structures. Additionally, the solvent effects were taken into consideration in all calculations using the SMD model. Fe=O bond energy was calculated assuming that Fe and O atoms were split into neutral atoms.

For periodic calculation, projector-augmented wave (PAW) was used to describe the electron–ion interactions,^6^ and the generalized gradient approximation functional (GGA-PBE) was applied to describe the electron exchange and correlation.^7^ A cut-off energy of 450 eV and a k-point mesh (3 × 3 × 1) were selected. The energy and force converged to 10^−5^ eV and -0.03 eV/Å, respectively. Since GGA cannot correctly describe strongly correlated systems containing partially filled d subshells, the GGA+U method is applied to describe partially filled d orbitals by taking into account Coulomb and exchange corrections. We used correlation energy (U) of 5 eV and exchange energy (J) of 1 eV for Fe d orbitals. A vacuum slab of 25 Å in the Z-direction was chosen to avoid artificial interaction. The effect of van der Waals (vdW) interactions was included for weak interaction cases using the semiempirical correction scheme of Becke-Jonson damping, DFT-D3.^8^ For PMS adsorption, the adsorption energy (E_ads_) was determined by eq. S2.

E_ads_ = E_PMS/catalyst_ − (E_PMS_ + E_catalyst_) (eq. S2)

Where, E_PMS/catalyst_, E_PMS_, and E_catalyst_ represented the total energies of the PMS/catalyst system, isolated PMS and catalyst surface in the same slab, respectively.

**Text S10 Kinetic modeling**

The kinetic modeling for the homogeneous Fe^2+^/PMS system was established using the Kintecus Version 2021 Package 3, with the modeling reactions shown in Table S1 ([www.kintecus.com](http://www.kintecus.com)).^9^

**Text S11 Details for membrane-based filtration apparatus**

FePC or FePPC catalysts (25 mg) were dispersed in 200 mL deionized water and sonicated for 30 min. The mixture was then loaded onto mixed cellulose ester membrane by vacuum filtration, and then assembled into the filtration apparatus. The flow rates of PMS solution and ACV solution were both set at 1 mL/min. The normalization of treatment capacity was conducted via eq. S3^10^.

$\text{Q}_{\text{normalized}}\text{ = }\frac{\text{Q}_{\text{flux}}\text{ × }\text{c}_{\text{ACV}}\text{ }\text{× IP}_{\text{ACV}}\text{ × removal percentage}}{\text{c}_{\text{PMS}}\text{ × }\text{m}_{\text{catalyst}}}$ (eq. S3)

Where, Q_normalized_ represented normalized treatment efficiency (L·eV·day^−1^·g^−1^), Q_flux_ represented water flux (L/day), c_ACV_ represented ACV concentration (mol/L), c_PMS_ represented PMS concentration (mol/L), m_catalyst_ represented catalyst mass (g), and IP_ACV_ represented the ionization potential of ACV (6.1 eV).

**Figure S1** (a) Simulated concentration change of Fe^2+^, PMS and methyl phenyl sulfoxide (PMSO, a benchmark probe for Fe(IV)) in Fe^2+^/PMS system. (b) Simulated ROS evolution curves in Fe^2+^/PMS system. Simulation conditions: [PMS] = 0.2 mM, [Fe^2+^] = 0.2 mM, [PMSO] = 0.2 mM.

**Figure S2** Simulated Fe(IV) formation and consumption by different species in Fe^2+^/PMS system. Simulation conditions: [PMS] = 0.2 mM, [Fe^2+^] = 0.2 mM, [PMSO] = 0.2 mM.

**Figure S3** Simulated concentration change of Fe^2+^, PMS and PMSO in Fe^2+^/PMS system with different initial Fe^2+^ concentrations of (a) 0.05 mM, (b) 0.1 mM, (c) 0.4 mM, and (d) 0.8 mM. Other simulation conditions: [PMS] = 0.2 mM, [PMSO] = 0.2 mM.

**Figure S4** Practical PMSO_2_ generation amount and the corresponding valid Fe(IV) yield from PMS. Experimental conditions: [PMS] = 0.2 mM, [PMSO] = 0.2 mM, pH = 3.0

**Figure S5** Self-assembly mechanism of FePPC framework.

The synthesis of FePPC consists of two consecutive steps: during the first step four 1,2,4,5-tetracyanobenzene molecules quickly condensed into octacyanophthalocyanine with the assistance of center iron atom below 200 °C. When the temperature reached 400 °C, such an octacyanophthalocyanine acts as a monomer and self-assembled into the extended organic framework even without the catalysis of central metal atom.

**Figure S6** Top and side views of the AB stacking model of FePPC.

**Figure S7** Derivative thermogravimetry (DTG) curves of FePC and FePPC as a function of temperature.

**Figure S8** SEM images of FePC with different magnification.

**Figure S9** SEM images of FePPC with different magnification.

**Figure S10** Particle size distributions of FePC and FePPC.

**Figure S11** N_2_ adsorption and desorption isotherms of FePC and FePPC.

**Figure S12** FE-SEM-EDS results for FePC and FePPC.

**Figure S13** Aberration corrected TEM images and the corresponding element mapping of FePC.

**Figure S14** (a) FT-EXAFS fitting of the Fe K-edge of FePPC in R-space. (b) FT-EXAFS fitting of the Fe K-edge of FePPC in k-space.

**Figure S15** (a) N k-edge XANES spectra of FePC and FePPC. (b) Fe L-edge XANES spectra of FePC and FePPC.

**Figure S16** (a) C 1s XPS spectra of FePC and FePPC. (b) N 1s XPS spectra of FePC and FePPC.

**Figure S17** UV-vis spectra of FePC and FePPC.

**Figure S18** Comparison of the energy of different spin state of molecular FePC with different polymerization (or conjugation) degrees

**Figure S19** Illustration of the HOMO-LUMO energy distribution along with the increase of conjugation degrees.

**Figure S20** PMS residual amount in the presence and absence of ACV in (a) FePC/PMS and (b) FePPC/PMS systems. Experimental conditions: [PMS] = 0.2 mM, [FePC] = 0.1 g/L.

Notably, the PMS consumption of FePC/PMS system would remarkably decrease in the presence of high-concentration ACV, indicating that the presence of ACV prevented PMS from excessive decomposition.

**Figure S21** Formation of O_2_ in FePC/PMS and FePPC/PMS systems in the presence or absence of ACV, Experimental conditions: [PMS] = 5 mM, [catalysts] = 0.5 g/L, [ACV] = 1 mM.

**Figure S22** Effects of (a) catalyst dosage, (b) PMS dosage, and (c) initial ACV concentration on the ACV removal efficiency of FePC/PMS system. Effects of (d) catalyst dosage, (e) PMS dosage, and (f) initial ACV concentration on the ACV removal efficiency of FePPC/PMS system. Experimental conditions: [PMS] = 0.2 mM, [catalysts] = 0.1 g/L, [ACV] = 20 μM.

**Figure S23** Effects of initial pH on the ACV removal efficiency in a FePC/PMS and b FePPC/PMS systems. Experimental conditions: [PMS] = 0.2 mM, [catalysts] = 0.1 g/L, [ACV] = 20 μM.

**Figure S24** Zeta potential of FePC and FePPC as a function of solution pH.

**Figure S25** Cyclic degradation performance of FePC and FePPC. Experimental conditions: [PMS] = 0.2 mM, [catalysts] = 0.1 g/L, [ACV] = 20 μM.

**Figure S26** FT-IR spectra of (a) FePC and (b) FePPC before and after reactions.

**Figure S27** Comparison of the XRD patterns of FePC and FePPC after reactions

**Figure S28** SEM images of (a),(b) FePC and (c),(d) FePPC after reactions.

**Figure S29** (a) Fe K-edge XANES spectra of FePC before and after reactions. (b) Fe K-edge XANES spectra of FePPC before and after reactions.

**Figure S30** (a) C K-edge, (b) N K-edge, and (c) Fe L-edge XANES spectra of FePC before and after reactions. (d) C K-edge, (e) N K-edge, and (f) Fe L-edge XANES spectra of FePPC before and after reactions.

**Figure S31** (a) C 1s XPS, (b) N 1s XPS, and (c) Fe 2p XPS spectra of FePC before and after reactions. (d) C 1s XPS, (e) N 1s XPS, and (f) Fe 2p XPS spectra of FePPC before and after reactions.

**Figure S32** N_2_ adsorption and desorption isotherms of FePC and FePPC after reaction.

**Figure S33** Iron leaching concentration of FePC and FePPC (a) along with the reaction and (b) during cyclic experiments. Experimental conditions: [PMS] = 0.2 mM, [catalysts] = 0.1 g/L, [ACV] = 20 μM.

**Figure S34** Control experiments of the contribution of leached iron ions on ACV removal efficiency.

**Figure S35** Effects of different quenching reagents on the degradation performance of (a) FePC/PMS and (b) FePPC/PMS systems. Experimental conditions: [PMS] = 0.2 mM, [catalysts] = 0.1 g/L, [ACV] = 20 μM.

**Figure S36** Probe experiments using benzoic acid (BA), nitrobenzene (NB), and dimethyl phthalate (DMP) to exclude the contribution of sulfate radicals and hydroxyl radicals in (a) FePC/PMS and (b) FePPC/PMS systems. Experimental conditions: [PMS] = 0.2 mM, [catalysts] = 0.1 g/L, [BA] = [NB] = [DMP] = 20 μM.

**Figure S37** UV-vis spectra of SOSG after reactions in FePC/PMS, FePPC/PMS, and benchmark H_2_O_2_/NaOCl systems.

**Figure S38** Effects of pre-mixing on the degradation performance of (a) FePC/PMS and (b) FePPC/PMS systems. Experimental conditions: [PMS] = 0.2 mM, [catalysts] = 0.1 g/L, [ACV] = 20 μM.

**Figure S39** Effects of KSCN screening on the degradation performance of (a) FePC/PMS and (b) FePPC/PMS systems. Experimental conditions: [PMS] = 0.2 mM, [catalysts] = 0.1 g/L, [ACV] = 20 μM.

**Figure S40** Performance of H_2_PC towards PMS activation for ACV degradation. Experimental conditions: [PMS] = 0.2 mM, [H_2_PC] = 0.1 g/L, [ACV] = 20 μM.

**Figure S41** PMSO consumption, PMSO_2_ generation and the corresponding yield of PMSO_2_ as a function of time in (a) FePC/PMS and (b) FePPC/PMS systems. Experimental conditions: [PMS] = 0.2 mM, [catalysts] = 0.1 g/L, [PMSO] = 200 μM.

**Figure S42** Effects of catalyst dosage on (a) PMSO_2_ formation amount, (b) PMS consumption amount, and (c) valid Fe(IV) yield from PMS. Effects of PMS dosage on (c) PMSO_2_ formation amount, (d) PMS consumption amount, and (e) valid Fe(IV) yield from PMS. Experimental conditions: [PMS] = 0.2 mM, [catalysts] = 0.1 g/L, [PMSO] = 200 μM.

**Figure S43** (a) XRD patterns of FePPC-LP and FePC@CNT. (b) PMSO_2_ formation in FePPC-LP/PMS and FePC@CNT/PMS systems. (c) PMS consumption in FePPC-LP/PMS and FePC@CNT/PMS systems. (d) Valid Fe(IV) yield from PMS in FePPC-LP/PMS and FePC@CNT/PMS systems. Experimental conditions: [PMS] = 0.2 mM, [catalysts] = 0.1 g/L, [PMSO] = 200 μM.

**Figure S44** (a) PMS consumption, and (b) Fe(IV) conervsion ratio from PMS in different metal PC/PMS and metal PPC/PMS system. Experimental conditions: [PMS] = 0.2 mM, [catalysts] = 0.1 g/L, [PMSO] = 200 μM.

**Figure S45** OCP profiles with different PMS dosage. (a) FePC/PMS systems. (b) FePPC/PMS systems.

**Figure S46** Reaction pathways and energy profile diagram of the reaction of Fe(IV) generated on FePC and cis/trans-stilbene.

**Figure S47** Generation of cis-stilbene oxide and trans-stilbene oxide and the corresponding yields of stilbene oixde in the co-presence of cis-stilbene and trans-stilbene. (a) FePC/PMS system (b) FePPC/PMS system. Experimental conditions: [PMS] = 0.2 mM, [catalysts] = 0.1 g/L, [cis-stilbene] = [trans-stilbene] = 0.5 mM, solvent: [water] : [acetonitrile] = 20 : 80.

Clearly, cis-stilbene and trans-stilbene showed nearly completely conversion rate into the corresponding oxides suggesting the reliability of methods in comparing Fe(IV) reactivity.

**Figure S48** Degradation of cis-stilbene oxide and trans-stilbene oxide in (a) FePC/PMS and (b) FePPC/PMS systems. Experimental conditions: [PMS] = 0.2 mM, [catalysts] = 0.1 g/L, [cis-stilbene oxide] = [trans-stilbene oxide] = 0.1 mM, solvent: [water] : [acetonitrile] = 20 : 80.

**Figure S49** *In-situ* Raman spectra of FePC/PMS and FePPC/PMS system.

**Figure S50** Effects of D_2_O replacement on the removal performance of PMSO in (a) FePC/PMS and (b) FePPC/PMS systems. Effects of D_2_O replacement on the removal performance of ACV in (c) FePC/PMS and (d) FePPC/PMS systems. Note that the KIE of FePC was calculated based on the fast degradation stage.

The N−H bond in ACV is easily exchangeable, and thus upon dissolution in D_2_O, the N−H bond would be quickly deuterated. As a result, the kinetic isotope effects between N−H bond and Fe(IV) could be investigated by solvent exchange to D_2_O. Note that PMSO reacts with Fe(IV) via oxygen transfer pathway, and thus the kinetic isotope effects of PMSO degradation could be used to normalize the effects of D_2_O on solvent properties to obtain the effects of D_2_O on the oxidation process of Fe(IV) on ACV.

**Figure S51** Comparison of the proportion of generated ^16^O^16^O, ^16^O^18^O, and ^18^O^18^O in FePC/PMS and FePPC/PMS systems. Experimental conditions: [PMS] = 5 mM, [catalysts] = 0.5 g/L.

**Figure S52** the composition of their frontier orbitals containing π-3d-2p orbitals in diverse isolated FePC molecules with different conjugation degrees.

**Figure S53** (a) Comparison of the electron density at the bond critical point of different molecular FePCs. (b) Comparison of the mayer bond orders of different molecular FePCs.

**Figure S54** Change of Fe=O bonding energy with the conjugation degrees of FePC

**Figure S55** Compariosn of the different adsorption configurations of PMS on FePC and FePPC surface.

**Figure S56** Compariosn of the different adsorption configurations of PMS on Fe(IV)=OPC and Fe(IV)=OPPC surface.

**Figure S57** COHP plots of (a) Periodic FePC and (b) FePPC catalysts.

**Figure S58** Effects of different practical water samples on the degradation of ACV in (a) FePC/PMS and (b) FePPC/PMS systems.

**Figure S59** Degradation pathways of ACV in FePPC/PMS system.

**Figure S60** Iron leaching of FePC- and FePPC-based membrane filtration systems.

**Table S1** Kinetic models of Fe^2+^/PMS system.

| No. | Reactions | Rate constants | Reference |
| --- | --- | --- | --- |
| Fe(IV) formation | | | |
| 1 | Fe^2+^ + HSO_5_^−^ → Fe^3+^ + SO_4_^•−^ + OH^−^ | 2.1 × 10^4^ M^−1^ s^−1^ | ^11^ |
| 2 | Fe^2+^ + HSO_5_^−^ → FeO^4+^ + SO_4_^2−^ + H^+^ | 2.2 × 10^4^ M^−1^ s^−1^ | ^11^ |
| Fe(IV) consumption | | | |
| 3 | FeO^4+^ (+ H^+^) → Fe^3+^ + 1/4 O_2_ + 1/2 H_2_O | 0.07 s^−1^ | ^12^ |
| 4 | FeO^4+^ + Fe^2+^ → products | 9.0 × 10^4^ M^−1^ s^−1^ | ^11^ |
| 5 | FeO^4+^ + HSO_5_^−^ → Fe^3+^ + SO_5_^•−^ + OH^−^ | 2.8 × 10^5^ M^−1^ s^−1^ | ^11^ |
| 6 | FeO^4+^ + H_2_O_2_ → Fe^3+^ + H_2_O + O_2_ | 2.4 × 10^4^ M^−1^ s^−1^ | ^12^ |
| 7 | FeO^4+^ + SO_4_^•−^ → Fe^3+^ + SO_5_^•−^ | 1.2 × 10^6^ M^−1^ s^−1^ | ^11^ |
| 8 | FeO^4+^ + SO_4_^•−^ → Fe^3+^ + SO_5_^•−^ | 1.26 × 10^8^ s^−1^ |  |
| Radical chain reactions | | | |
| 9 | SO_4_^•−^ (+ H_2_O) → SO_4_^2−^ + HO^•^ + H^+^ | 9.3 s^−1^ | ^13^ |
| 10 | SO_4_^•−^ + HO^•^ → HSO_5_^−^ | 1.0 × 10^10^ M^−1^ s^−1^ | ^13^ |
| 11 | SO_4_^•−^ + OH^−^ → SO_4_^2−^ + HO^•^ | 7.0 × 10^7^ M^−1^ s^−1^ | ^14^ |
| 12 | HO^•^ + HSO_5_^−^ →SO_5_^•−^ + H_2_O | 1.7 × 10^7^ M^−1^ s^−1^ | ^14^ |
| 13 | SO_4_^•−^ + HSO_5_^−^ →SO_5_^•−^ + SO_4_^2−^ + H^+^ | 1.0 × 10^6^ M^−1^ s^−1^ | ^13^ |
| 14 | SO_4_^•−^ + SO_4_^•−^ → S_2_O_8_^2−^ | 4.5 × 10^8^ M^−1^ s^−1^ | ^11^ |
| 15 | SO_5_^•−^ + SO_5_^•−^ → S_2_O_8_^2−^ + O_2_ | 2.1 × 10^8^ M^−1^ s^−1^ | ^13^ |
| 16 | SO_5_^•−^ + SO_5_^•−^ → 2SO_4_^•−^ + O_2_ | 2.2 × 10^8^ M^−1^ s^−1^ | ^11^ |
| 17 | Fe^2+^ + SO_5_^•−^ + H^+^ → Fe^3+^ + HSO_5_^−^ | 4.3 × 10^7^ M^−1^ s^−1^ | ^11^ |
| 18 | Fe^2+^ + HO^•^ → Fe^3+^ + OH^−^ | 2.78 × 10^8^ M^−1^ s^−1^ | ^13^ |
| 19 | Fe^2+^ + SO_4_^•−^ → Fe^3+^ + SO_4_^2−^ | 4.6 × 10^9^ M^−1^ s^−1^ | ^13^ |
| 20 | S_2_O_8_^2−^ + HO^•^ →S_2_O_8_^•−^ + OH^−^ | 1.2 × 10^7^ M^−1^ s^−1^ | ^13^ |
| 21 | S_2_O_8_^2−^ + SO_4_^•−^ → S_2_O_8_^•−^ + SO_4_^2−^ | 6.1 × 10^5^ M^−1^ s^−1^ | ^13^ |
| 22 | HO^•^ + HO^•^ →H_2_O_2_ | 5.5 × 10^9^ M^−1^ s^−1^ | ^13^ |
| 23 | H^+^ + OH^−^ → H_2_O | 1.0 × 10^11^ M^−1^ s^−1^ | ^15^ |
| 24 | H_2_O → H^+^ + OH^−^ | (1.81 ± 0.18) × 10^10^ M^−1^ s^−1^ | ^15^ |
| 25 | H^+^ + O_2_^•−^ → HO_2_^•^ | 5.0 × 10^10^ M^−1^ s^−1^ | ^13^ |
| 26 | HO_2_^•^ → H^+^ + O_2_^•−^ | 7.9 × 10^5^ M^−1^ s^−1^ | ^11^ |
| 27 | HO^•^ + H_2_O_2_ → H_2_O + HO_2_^•^ | 2.7 × 10^7^ M^−1^ s^−1^ | ^13^ |
| 28 | HO^•^ + HO_2_^•^ → H_2_O + O_2_ | 1.0 × 10^10^ M^−1^ s^−1^ | ^13^ |
| 29 | H_2_O → H^+^ + OH^−^ | (1.81 ± 0.18) × 10^10^ M^−1^ s^−1^ | ^13^ |
| Substrate reactions | | | |
| 30 | HO^•^ + PMSO → products | 3.61 × 10^9^ M^−1^ s^−1^ | ^13^ |
| 31 | SO_4_^•−^ + PMSO → products | 3.17 × 10^8^ M^−1^ s^−1^ | ^11^ |
| 32 | FeO^4+^ + PMSO → products | 1.1 × 10^5^ M^−1^ s^−1^ | ^12^ |

Note that species in parentheses were written to keep equations balanced but were not taken into account in the kinetic model. Besides, the model was established at pH 1 due to the availability of several pivotal kinetic constants.

**Table S2** XAS fitting results of FePPC ($\text{S}_{\text{0}}^{\text{2}}\text{ = 0.909}$).

|  | Path | Degeneracy | ^a^R (Å) | ^b^10^^3^ × σ^2^ (Å^2^) | ^c^ΔE_0_ (eV) | R factor (%) |
| --- | --- | --- | --- | --- | --- | --- |
| Fe foil | Fe−Fe path | 8 | 2.47 ± 0.01 | 7.11 | 6.244 | 1.11 |
|  | Fe−Fe path | 6 | 2.84 ± 0.03 | 7.05 | 6.244 |  |
| FePPC | Fe−N path | 4 | 1.92 ± 0.01 | 5.34 | 6.244 | 0.24 |
|  | Fe−C path | 8 | 2.96 ± 0.02 | 6.55 | 6.244 |  |
|  | Fe−N−C path | 12 | 3.13 ± 0.07 | 3.02 | 6.244 |  |

^a^R, the distance between absorber and backscatter atoms: ^b^σ. Debye-Waller factor to account for both thermal and structural disorders, ^c^ΔE_0_, inner potential correction; R factor indicates the goodness of the fitting. $\text{S}_{\text{0}}^{\text{2}}$ was fixed as 0.909, according to the experimental EXAFS fit of Fe foil by fixing CN as the known crystallographic value. Fitting range: 3 ≤k (Å^−1^)≤ 15 and 1.5 ≤ R (Å) ≤ 4 Fe foil); 3.0 ≤k (/Á)≤ 11.0 and 1.0 ≤ R (A)≤ 3 (FePPC)

**Table S3** Parameters derived from the fittings of Mössbauer spectra.

|  | IS mm s^−1^ | QS mm s^−1^ | Assignment |
| --- | --- | --- | --- |
| FePC | 0.364 | 2.403 | Intermediate spin Fe(II)-N_4_ |
| FePPC | 0.147 | 2.08 | Intermediate spin Fe(II)-N_4_ |

**Table S4** Fitting results of Nyquist plots.

|  | Rs (Ω) | Rct (Ω) | CPE (F) | W_R_ (Ω) |
| --- | --- | --- | --- | --- |
| FePC | 6 | 128.3 | 4.67E-09 | 1.6 |
| FePPC | 7.5 | 120 | 4.02E-09 | 2.7 |

**Table S5** Comparison of ACV degradation performance with references.

| Catalysts | ACV concentration (μM) | Oxidant concentration (mM) | Catalyst dosage (g/L) | Apparent reaction constants (min^−1^) | Normalized *k*_app_ (eV g L^−1^ min^−1^) | Ratio of kapp after cyclic experiments compared with initial value | Other reaction condition | References |
| --- | --- | --- | --- | --- | --- | --- | --- | --- |
| NH_2_-MIL-53(Fe)-650°C | 44.4 | 0.65 | 0.1 | 0.1377 | 0.57 | 0.32 | pH=3.6, room temperature | ^16^ |
| Fe-N_5_ single-atom catalysts | 8.88 | 0.5 | 0.15 | 0.444 | 0.32 | 1 | 25°C | ^17^ |
| Fe-N/C-C single-atom catalysts | 44.4 | 3 | 0.05 | 0.138 | 0.25 | 0.183 | 25°C | ^18^ |
| FeN_x_ single-atom catalysts | 44.4 | 0.5 | 0.1 | 0.178 | 0.96 | 0.04 6 | 25°C | ^19^ |
| Co@C-NO_3_ | 44.4 | 3 | 0.1 | 0.0318 | 0.03 | 0.035 | 25°C | ^20^ |
| s-TiO_2_@CaTiO_3_ | 11.1 | 0.2 | 0.4 | 0.0553 | 0.05 | 1 | Visible light irradiation, 25°C | ^21^ |
| macroporous carbon nitride supported Co single atoms | 44.4 | 0.5 | 0.2 | 0.045 | 0.12 | 0.81 | Visible light irradiation, 25°C | ^22^ |
| FePPC | 20 | 0.2 | 0.1 | 0.11 | 0.67 | 1.1 | 25°C | This work |

**Table S6** HPLC conditions for different contaminants.

| Contaminants | Flow rate (mL/min) | Mobile phase | Wavelength (nm) | Column temperature (°C) |
| --- | --- | --- | --- | --- |
| Acyclovir | 0.9 | 0.1% acetic acid : acetonitrile = 40 : 60 | 264 | 30 |
| Nitrobeneze | 0.8 | 0.1% acetic acid : methanol = 50 : 50 | 262 | 30 |
| Dimethyl phthalate | 0.8 | 0.1% acetic acid: acetonitrile = 40 : 60 | 225 | 30 |
| Methyl phenyl sulfone | 1.0 | 0.1% acetic acid : acetonitrile = 70 : 30 | 215 | 30 |
| Methyl phenyl sulfoxide | 1.0 | 0.1% acetic acid : acetonitrile = 70 : 30 | 215 | 30 |
| 4-chlorophenol | 1.0 | 0.1% acetic acid : acetonitrile = 40 : 60 | 280 | 30 |
| Carbamazepine | 0.8 | 0.1% acetic acid : acetonitrile = 40 : 60 | 286 | 30 |
| Sulfamethazole | 1.0 | 0.1% acetic acid : acetonitrile = 40 : 60 | 264 | 30 |
| Propranolol | 1.0 | 0.1% acetic acid : acetonitrile = 70 : 30 | 280 | 30 |
| Benzaldehyde | 1.0 | 0.1% acetic acid : acetonitrile = 40 : 60 | 264 | 30 |
| Benzoic acid | 0.9 | 0.1% H_3_PO_4_: acetonitrile = 40 : 60 | 230 | 30 |

**Table S7** Transformation product (TP) information of ACV (positive ion mode).

| TP | Molecular Formula | *m/z* | Molecular structures | Retention time (min) |
| --- | --- | --- | --- | --- |
| Acyclovir | C_8_H_11_N_5_O_3_ | [M+H]^+^ = 226.0948 |  | 1.352 |
| TP229 | C_7_H_11_N_5_O_4_ | [M+H]^+^ = 230.0882 |  | 1.189 |
| TP213 | C_7_H_11_N_5_O_3_ | [M+H]^+^ = 214.0946 |  | 1.199 |
| TP151 | C_5_H_5_N_5_O | [M+H]^+^ = 152.0570 |  | 1.346 |
| TP113 | C_5_H_11_N_3_ | [M+H]^+^ = 114.0923 |  | 1.908 |

**References:**

(1).	Huang, B.; Ren, X.; Zhao, J.; Wu, Z.; Wang, X.; Song, X.; Li, X.; Liu, B.; Xiong, Z.; Lai, B., Modulating Electronic Structure Engineering of Atomically Dispersed Cobalt Catalyst in Fenton-like Reaction for Efficient Degradation of Organic Pollutants. *Environ. Sci. Technol.* **2023**.

(2).	Frisch, M. J.; Trucks, G. W.; Schlegel, H. B.; Scuseria, G. E.; Robb, M. A.; Cheeseman, J. R.; Scalmani, G.; Barone, V.; Petersson, G. A.; Nakatsuji, H.; Li, X.; Caricato, M.; Marenich, A. V.; Bloino, J.; Janesko, B. G.; Gomperts, R.; Mennucci, B.; Hratchian, H. P.; Ortiz, J. V.; Izmaylov, A. F.; Sonnenberg, J. L.; Williams; Ding, F.; Lipparini, F.; Egidi, F.; Goings, J.; Peng, B.; Petrone, A.; Henderson, T.; Ranasinghe, D.; Zakrzewski, V. G.; Gao, J.; Rega, N.; Zheng, G.; Liang, W.; Hada, M.; Ehara, M.; Toyota, K.; Fukuda, R.; Hasegawa, J.; Ishida, M.; Nakajima, T.; Honda, Y.; Kitao, O.; Nakai, H.; Vreven, T.; Throssell, K.; Montgomery Jr., J. A.; Peralta, J. E.; Ogliaro, F.; Bearpark, M. J.; Heyd, J. J.; Brothers, E. N.; Kudin, K. N.; Staroverov, V. N.; Keith, T. A.; Kobayashi, R.; Normand, J.; Raghavachari, K.; Rendell, A. P.; Burant, J. C.; Iyengar, S. S.; Tomasi, J.; Cossi, M.; Millam, J. M.; Klene, M.; Adamo, C.; Cammi, R.; Ochterski, J. W.; Martin, R. L.; Morokuma, K.; Farkas, O.; Foresman, J. B.; Fox, D. J. *Gaussian 16 Rev. C.01*, Wallingford, CT, 2016.

(3).	Lu, T.; Chen, F., Multiwfn: A multifunctional wavefunction analyzer. *J. Comput. Chem.* **2012,** *33*, (5), 580-592.

(4).	Kresse, G.; Hafner, J., Ab initio molecular dynamics for liquid metals. *Phys. Rev. B* **1993,** *47*, (1), 558-561.

(5).	Kresse, G.; Furthmüller, J., Efficient iterative schemes for ab initio total-energy calculations using a plane-wave basis set. *Phys. Rev. B* **1996,** *54*, (16), 11169-11186.

(6).	Blöchl, P. E., Projector augmented-wave method. *Phys. Rev. B* **1994,** *50*, (24), 17953-17979.

(7).	Perdew, J. P.; Burke, K.; Ernzerhof, M., Generalized Gradient Approximation Made Simple. *Phys Rev Lett* **1996,** *77*, (18), 3865-3868.

(8).	Grimme, S.; Antony, J.; Ehrlich, S.; Krieg, H., A consistent and accurate ab initio parametrization of density functional dispersion correction (DFT-D) for the 94 elements H-Pu. *J Chem Phys* **2010,** *132*, (15), 154104.

(9).	Ianni, J., Kintecus Version 2021 Package 3. [*http://www.kintecus.com*](http://www.kintecus.com) **2022**.

(10).	Zhou, H.; Ren, W.; Wang, S.; Duan, X., Best practices for advanced oxidation processes. *Nat. Sustain.* **2025,** *8*, (12), 1428-1431.

(11).	Dong, H.; Xu, Q.; Lian, L.; Li, Y.; Wang, S.; Li, C.; Guan, X., Degradation of Organic Contaminants in the Fe(II)/Peroxymonosulfate Process under Acidic Conditions: The Overlooked Rapid Oxidation Stage. *Environ. Sci. Technol.* **2021,** *55*, (22), 15390-15399.

(12).	Wang, Z.; Shin, J.; Lee, J.; Lee, C.; Lee, Y., Formation and Decay of Aqueous Fe(IV) during Fe(II) Ozonation under Acidic Conditions: Kinetics and Mechanistic Insights into pH-Dependent Behavior. *Environ. Sci. Technol.* **2025,** *59*, (48), 26217–26228.

(13).	Rao, D.; Dong, H.; Niu, M.; Wang, X.; Qiao, J.; Sun, Y.; Guan, X., Mechanistic Insights into the Markedly Decreased Oxidation Capacity of the Fe(II)/S2O82– Process with Increasing pH. *Environ. Sci. Technol.* **2022,** *56*, (18), 13131-13141.

(14).	Wang, A.; Hua, Z.; Chen, C.; Wei, W.; Huang, B.; Hou, S.; Li, X.; Fang, J., Radical chemistry and PPCP degradation in the UV/peroxydisulfate process in the presence of chloride at freshwater levels. *Chem. Eng. J.* **2021,** *426*, 131276.

(15).	Zhou, H.; Zhong, S.; Chen, J.; Ren, S.; Ren, W.; Lai, B.; Guan, X.; Ma, T.; Wang, S.; Duan, X., Overlooked Complexation and Competition Effects of Phenolic Contaminants in a Mn(II)/Nitrilotriacetic Acid/Peroxymonosulfate System: Inhibited Generation of Primary and Secondary High-Valent Manganese Species. *Environ. Sci. Technol.* **2024,** *58*, (42), 19080-19089.

(16).	Liu, C.; Wang, Y.; Zhang, Y.; Li, R.; Meng, W.; Song, Z.; Qi, F.; Xu, B.; Chu, W.; Yuan, D.; Yu, B., Enhancement of Fe@porous carbon to be an efficient mediator for peroxymonosulfate activation for oxidation of organic contaminants: Incorporation NH2-group into structure of its MOF precursor. *Chem. Eng. J.* **2018,** *354*, 835-848.

(17).	Song, X.; Shi, Y.; Wu, Z.; Huang, B.; Wang, X.; Zhang, H.; Zhou, P.; Liu, W.; Pan, Z.; Xiong, Z.; Lai, B., Unraveling the discriminative mechanisms for peroxy activation via atomically dispersed Fe-N5 sites for tunable water decontamination. *Appl. Catal. B: Environ.* **2024,** *340*, 123240.

(18).	Ren, S.; Wang, Y.; Shi, L.; Xu, X.; Zhong, S.; Hu, K.; Zhou, H.; Zhu, Z.-S.; Zhou, P.; Tian, W.; Zuo, J.; Yi, J.; Guan, X.; Duan, X.; Wang, S., Transforming Plastics to Single Atom Catalysts for Peroxymonosulfate Activation: Axial Chloride Coordination Intensified Electron Transfer Pathway. *Adv. Mater.* **2025,** *37*, (8), 2415339.

(19).	Li, Z.; Wang, Z.; Dai, L.; Ma, J.; Liu, X. e., Highly effective catalytic degradation of bisphenol A through activation of peroxymonosulfate by an Fe-Nx structure anchored on novel lignin-based graphitic biochar: Electron transfer mechanism. *International Journal of Biological Macromolecules* **2025,** *286*, 138322.

(20).	Tian, Z.; Chen, Q.; Ren, S.; Zhang, H.; Tian, W.; Sun, H.; Wang, S., Effects of cobalt salts on biomass conversion to functional carbon-based catalysts for peroxymonosulfate activation. *Chem. Eng. J.* **2023,** *469*, 143856.

(21).	Zhang, J.; Yan, Y.; Armutlulu, A.; Wei, K.; Wu, J.; Lai, B.; Xie, R., Assembling a durable homologous-like heterojunction s-TiO2@CaTiO3 for photodegradation of atrazine through boosting charge migration with peroxymonosulfate reinforcement. *Chem. Eng. J.* **2024,** *490*, 151300.

(22).	Lin, J.; Jiang, L.; Tian, W.; Yang, Y.; Duan, X.; Jiao, Y.; Zhang, H.; Wang, S., The structure-dependent mechanism of single-atom cobalt on macroporous carbon nitride in (photo-)Fenton-like reactions. *J. Mater. Chem. A* **2023,** *11*, (25), 13653-13664.
